# Integrated Multi-Omics Analysis and Experimental Validation Identify Acetylation-Related Genes as Potential Regulators in Osteoarthritis

**DOI:** 10.3390/biomedicines14081806

**Published:** 2026-08-11

**Authors:** Qiaojun Huang, Xiaoyi Zhao, Dianbo Long, Ming Li, Yiyi Jiang, Hengyi Diao, Weishen Chen, Fangang Meng

**Affiliations:** 1 Department of Sports Medicine, The First Affiliated Hospital of Sun Yat-sen University, Guangzhou 510080, China; huangqj23@mail2.sysu.edu.cn (Q.H.);; 2 Department of Joint Surgery, The First Affiliated Hospital of Sun Yat-sen University, Guangzhou 510080, China

**Keywords:** osteoarthritis, histone acetylation, bioinformatics, gene expression, immunoinfiltration

## Abstract

**Background:** Osteoarthritis (OA) is a prevalent degenerative joint disease with a complex molecular basis. This study aims to identify key molecules involved in OA pathogenesis, focusing on the role of acetylation-related gene expression. **Methods:** Public microarray datasets GSE82107 and GSE169077 were integrated to construct a differential expression landscape between OA patients and healthy controls. Acetylation-linked differentially expressed genes (acetylation-DEGs, ARDEGs) were extracted by intersecting DEGs with a curated set of acetyltransferases, deacetylases and acetylation substrates. A protein–protein interaction (PPI) network was built and subjected to LASSO-penalized regression to prioritise hub genes. Gene Ontology (GO), Kyoto Encyclopaedia of Genes and Genomes (KEGG) and Gene Set Variation Analysis (GSVA) were performed to characterize biological themes. Immune infiltration was quantified with CIBERSORTx and single-sample Gene Set Enrichment Analysis (ssGSEA). Single-cell RNA-seq data (GSE216651) were employed for orthogonal validation. For experimental corroboration, synovial tissue was collected from OA patients undergoing arthroplasty; mRNA and protein levels of hub genes were determined by qRT-PCR, Western blot and immunofluorescence. The destabilisation of the medial meniscus (DMM) mouse model was used for in vivo verification. **Results:** Twenty-one high-confidence ARDEGs were identified. Analysis of the PPI network yielded ten hub nodes, six of which (*EGR1*, *PFKFB3*, *HDAC4*, *MMP13*, *PDK4* and *ACADL*) retained non-zero coefficients in the least absolute shrinkage and selection operator (LASSO) model. Enrichment analyses implicated these genes in embryonic development, collagen-containing extracellular matrix remodeling and PI3K–Akt signaling. Immune infiltration analysis showed potential differences in immune cell abundance between OA and healthy controls. Single-cell dataset analysis verified the expression patterns of key genes in different cell types. Concordant dysregulation of *EGR1*, *PFKFB3*, *HDAC4*, *MMP13* and *PDK4* was observed at both mRNA and protein levels in human OA synovium and DMM mouse joints. **Conclusion:** This comprehensive analysis identified acetylation-related genes and analyzed their potential biological roles in OA. The identified ARDEGs may provide new insights into OA diagnosis and treatment.

## 1. Introduction

Osteoarthritis (OA), a widespread degenerative condition affecting joints, is the leading cause of joint destruction, pain, and disability worldwide, imposing substantial burdens on patients, families, and society [1]. From 1990 to 2020, the number of patients with OA increased significantly from approximately 256 to 595 million globally, reflecting an increase of 132%, accounting for 7.6% of the global population [2]. OA—a low-grade, nonspecific inflammatory disease affecting the entire joint—is characterized by synovitis, cartilage erosion, subchondral bone defects, and infrapatellar fat pad damage [3]. Notably, the infrapatellar fat pad (IFP) and synovial membrane (SM) have been increasingly recognized as forming an anatomo-functional unit (AFU) [4]. The IFP, a fibro-adipose tissue in close contact with the SM, serves as the insertion site for synovial plicae and actively participates in inflammatory mediator release during OA progression [5]. This anatomical and functional interdependence underscores the importance of studying both tissues in understanding OA pathophysiology. However, which of these processes are causal and which are secondary reactions to joint damage remains unclear [6]. The risk factors for OA include age, body weight, and sex, among others. With the advances in molecular biology techniques in recent years, researchers have gradually unveiled the complex molecular mechanisms underlying OA, with an increasing focus on molecular biological changes, including gene expression regulation, signaling pathways, and apoptosis mechanisms [7]. Through bioinformatics analysis, Wang et al. revealed the potential role of genes including cluster of differentiation 4 (*CD4*), selectin L (*SELL*), integrin subunit beta 2 (*ITGB2*), and cluster of differentiation 52 (*CD52*) in OA pathogenesis. These findings were further confirmed using quantitative polymerase chain reaction and immunohistochemistry [8]. He et al. combined bioinformatic analysis with experimental validation to identify ferroptosis- and cuproptosis-related genes, including cyclin-dependent kinase inhibitor 1A (*CDKN1A*), frizzled class receptor 7 (*FZD7*), and solute carrier family 39 member 14 (*SLC39A14*), as potential diagnostic biomarkers for OA [9].

Protein acetylation is a key post-translational modification mechanism. These post-translational modifications, including acetylation and ubiquitination, perform essential functions in influencing gene expression. Notably, the acetylation process of histones and non-histone proteins, primarily lysine residues, profoundly impacts the regulation of gene expression and various biological functions [10,11,12]. Histone acetyltransferases and histone deacetylases (HDACs) work together to fine-tune protein stability and transcriptional activity by regulating the acetylation status of non-histone proteins (e.g., transcription factors) [10,12,13].

Protein acetylation is essential for modulating diverse cellular processes via multiple mechanisms. Its primary functions in cellular regulation include influencing gene expression, modulating enzyme activity, controlling cell cycle progression, facilitating signal transduction, and mediating protein–protein interactions [14,15]. The acetylation landscape of joint tissues is sculpted by the opposing activities of histone acetyltransferases (HATs), histone deacetylases (HDACs), and sirtuins (SIRT1–7). HDACs, encompassing class I (HDAC1–3, 8), class IIa (HDAC4, 5, 7, 9), class IIb (HDAC6, 10), and class IV (HDAC11), remove acetyl marks and have been implicated in extracellular matrix (ECM) stiffening, chondrocyte senescence, and inflammatory resolution [16]. Sirtuins, as NAD+-dependent deacetylases, integrate metabolic status with epigenetic control; specifically, SIRT1-mediated deacetylation of Akt enhances chondrocyte survival and autophagy, whereas SIRT6 attenuates nuclear factor-κB (NF-κB)-driven inflammatory signaling [16]. Conversely, HATs such as p300/CBP deposit acetyl groups to activate transcriptional programs underlying cartilage degradation and immune dysregulation. The delicate equilibrium among these enzyme families underscores the translational potential of targeted epigenetic interventions for OA [16]. Among the numerous factors implicated in OA pathogenesis, post-translational modification of histone proteins through acetylation has emerged as a pivotal mechanism. This process modulates chromatin accessibility, thereby influencing the gene expression patterns that are integral to the inflammatory and immune responses associated with OA. Consequently, acetylation has garnered considerable attention in OA research, underscoring its potential as a therapeutic target and biomarker of OA. Recent evidence underscores that acetylation modifications serve as pivotal regulatory switches across the whole-joint milieu in OA. Histone acetylation modulates chromatin accessibility at the promoters of inflammatory and catabolic genes, thereby governing the expression of Interleukin-1β (IL-1β), tumor necrosis factor-α (TNF-α), and matrix metalloproteinases in cartilage, synovium, and subchondral bone [17]. Equally important, non-histone protein acetylation affects the stability, subcellular localization, and enzymatic activity of diverse substrates, including transcription factors (e.g., NF-κB, p53, and RUNX2), metabolic enzymes, and cytoskeletal proteins, implicating acetylation in the coordinated regulation of chondrocyte hypertrophy, synovial fibroblast activation, and osteoclastogenesis [17].

However, the specific mechanisms of acetylation-related genes (ARGs) in OA development and progression remain understudied. This research sought to address this gap by offering novel insights into OA pathogenesis through a detailed analysis of these critical pathways and gene expression, thereby establishing a theoretical basis for OA diagnosis and treatment. Our study focuses on gene expression patterns of acetylation-related genes. These findings offer hypotheses regarding the molecular mechanisms underlying OA and suggest candidate targets for future therapeutic strategies, pending functional validation of acetylation-dependent regulation and causal roles.

## 2. Materials and Methods

### 2.1. Data Source

Using the R package (v4.5) GEOquery [18], we retrieved human OA-related mRNA datasets GSE82107 [19] and GSE169077 from the Gene Expression Omnibus (GEO) database [20]. GSE82107, based on the GPL570 platform, comprises 10 end-stage OA synovial and 7 synovial samples from individuals without articular disease. GSE169077, based on the GPL96 platform, comprises five normal and six OA cartilage samples. The probe names in the datasets were annotated using the corresponding GPL platform gene annotation package in R. Single-cell data were from GSE216651. This study collected IPFP/synovial cells from 4 healthy donors and 5 OA patients and analyzed them via scRNA-seq [21]. The following inclusion criteria were applied: (1) the dataset contained gene expression profiling data from human OA samples and corresponding healthy controls; (2) the tissue type was synovial membrane or articular cartilage; (3) the platform was an Affymetrix microarray with available annotation packages in R; (4) the sample size was ≥5 per group to ensure statistical power; and (5) the study provided sufficient clinical information for disease classification. Datasets were excluded if: (1) they involved drug-treated or post-surgical samples that might confound the intrinsic OA gene expression signature; (2) they lacked proper control groups; or (3) they were derived from non-human organisms.

The GeneCards database, a comprehensive resource for human gene information [22], was searched using the keyword “acetylation” with a relevance score filter of >1. The search results yielded 4537 genes implicated in acetylation. Additionally, we searched the UniProt database, a global repository of protein sequences and annotations [23], for proteins associated with acetylation and deacetylation and identified 79 pertinent genes. Simultaneously, we also explored the Molecular Signatures Database (MSigDB) [24] using “acetylation” as the key term. The results revealed 48 genes related to acetylation from the GOBP_INTERNAL_PROTEIN_AMINO_ACID_ACETYLATION, GOBP_N_TERMINAL_PROTEIN_AMINO_ACID_ACETYLATION, GOBP_PEPTIDYL_LYSINE_ACETYLATION, and GOBP_PROTEIN_DEACETYLATION datasets. Furthermore, we included an additional 230 genes associated with acetylation in the literature and incorporated them into a database. By consolidating these genes from four distinct sources and eliminating redundancies, we assembled a comprehensive catalog of 4592 unique acetylation-associated genes.

### 2.2. Differential Expression Analysis

We initially employed the R package limma to merge the GSE82107 and GSE169077 datasets to investigate the biological processes and signaling pathways associated with OA [25]. We used the ComBat method from the R package sva to mitigate batch effects within datasets and constructed a unified GEO dataset [26]. Effectiveness of the correction was assessed by examining boxplots of the datasets pre- and post-correction.

After classifying the merged dataset into OA and control groups, differential expression analysis identified 80 significant differentially expressed genes (DEGs) based on the criteria |logFC| > 1 and *p*-adj < 0.05. Volcano plots and a heatmap of these DEGs were then constructed using the ggplot2 and pheatmap packages in R, respectively, to visualize the expression patterns of the DEGs. The Venn diagram intersection of the 80 significant DEGs with 4592 ARGs revealed 21 acetylation-related differentially expressed genes (ARDEGs) in OA.

### 2.3. Protein–Protein Interaction (PPI) Network

The associations and interactions between proteins can be graphically represented using a PPI network constructed from predicted and known interactions in the Search Tool for the Retrieval of Interacting Genes/Proteins (STRING) database [27]. Genes, particularly those regulating the same biological processes, often exhibit interconnectivity. Therefore, we constructed a PPI network to elucidate interactions among ARDEGs.

We utilized the STRING database, specifying *Homo sapiens* as the species of interest and setting a minimum required interaction score threshold of 0.4 to build a PPI network associated with ARDEGs. Cytoscape software (v3.10) was used for network visualization to enhance our understanding of these network structures [28]. Furthermore, we used the cytoHubba plugin within Cytoscape for topological analysis to identify key nodes in the PPI network [29]. Subsequently, the top 10 genes, which were considered the hub genes, were identified. PPI reveal functional relationships and regulatory hierarchies among acetylation-related genes. Genes participating in the same biological pathways often exhibit physical interactions, making PPI networks powerful tools for identifying functionally coherent gene modules.

### 2.4. Correlation Analysis of Hub Genes

We used the Spearman rank correlation algorithm to calculate the correlation coefficients of expression levels among hub genes. The correlation matrix between these genes was visualized using a correlation heatmap. To further elucidate the specific expression relationships between the pairs of hub genes with significant correlations, we selected the gene pairs with the highest correlation values and visually inspected their expression levels using scatter plots. Concurrently, for the scatter plot data of each gene pair, the correlation coefficient (r) and its corresponding *p*-value were calculated to quantitatively assess the strength and statistical significance of the correlation.

### 2.5. Least Absolute Shrinkage and Selection Operator (LASSO) Regression and Receiver Operating Characteristic (ROC) Curve Analyses

LASSO regression analysis is particularly suitable for models with many covariates. Its key advantage lies in enhancing the predictive accuracy of models by penalizing the absolute values of the regression coefficients, thereby performing variable selection and coefficient shrinkage [30]. To identify key hub genes, we conducted LASSO regression analysis on the 10 genes identified in the PPI network using the glmnet package in R. Furthermore, we used boxplots to compare gene expression levels between OA and normal samples. Additionally, we employed ROC curves to evaluate the efficacy of these hub genes in distinguishing healthy from OA samples. Given the high dimensionality of gene expression data and potential multicollinearity among hub genes, LASSO regression was employed to perform variable selection and coefficient shrinkage, identifying the minimal gene set with optimal diagnostic performance.

### 2.6. Gene Ontology (GO), Kyoto Encyclopedia of Genes and Genomes (KEGG), and Gene Set Variation Analysis (GSVA)

GO analysis is widely applied for comprehensive functional enrichment studies encompassing three dimensions: biological processes (BPs), molecular functions (MFs), and cellular components (CCs) [31]. KEGG is a well-known database resource that provides detailed information on genomics, biological processes, diseases, and drugs [32]. In this study, we employed the R package clusterProfiler for GO and KEGG annotation analyses of the key hub genes [33]. Additionally, we utilized the R package Pathview to visualize the metabolic pathway maps associated with enrichment analysis. GSVA is an unsupervised nonparametric method designed to assess transcriptome gene set enrichment [34]. We used the “Hallmark.all.v2022.1.Hs.symbols” gene set from the MSigDB as a reference set. To evaluate enrichment in normal and OA synovial samples, we scored the HALLMARK pathways using the R package GSVA. GO, KEGG and GSVA analyses were conducted to systematically characterize the biological processes, cellular components, molecular functions, and signaling pathways associated with acetylation-related DEGs, thereby elucidating their mechanistic involvement in OA.

### 2.7. Immune Infiltration Analysis

CIBERSORTx utilizes linear support vector regression to deconvolute transcriptomic expression matrices, thereby estimating the quantity and composition of immune cells across various cell populations. By integrating the gene expression matrix from our combined dataset with the LM22 gene signature matrix, we determined the abundance and distribution of immune cell infiltration. We merged the gene expression matrix with the LM22 signature matrix by uploading the data to the CIBERSORTx platform to construct an immune cell infiltration matrix. Within this matrix, data points with immune cell enrichment scores of ≤0 were excluded to ensure the scientific validity and accuracy of our findings. Stacked bar charts were used to display the proportions of immune cell infiltration among the different groups within our combined dataset. Finally, we combined the expression matrix of the key genes and generated a heatmap to illustrate the relationships between key gene expression and immune cell infiltration levels within the integrated dataset. Inter-group differences in immune cell proportions were compared using Student’s t-test, and *p*-values were adjusted for multiple testing across the 22 cell types using the Benjamini–Hochberg (FDR) method.

Single-sample gene set enrichment analysis (ssGSEA) is an extension of the GSEA method widely used in bioinformatics studies on immune infiltration [32]. We assessed the enrichment scores for 28 immune cell types in normal and OA synovial samples using the R package GSVA and presented the results using the R package vioplot. Acetylation modifications critically regulate immune cell function, chromatin accessibility in immune cells, and inflammatory gene expression. Therefore, characterizing immune cell composition and its relationship with acetylation-related hub genes is essential for understanding their pathophysiological roles in OA.

### 2.8. Validation of Single-Cell Datasets

The single-cell dataset GSE216651 profiles IPFP/synovial cells from four healthy donors and five OA patients [21]. Single-cell transcriptomic analyses are performed. Data preprocessing, quality control, normalization, and dimensional reduction clustering are done via the R package Seurat [35]. The R package Harmonic integrates data differences across samples. Then, the SingleR program names single-cell groups by combining literature-based signature genes with manual annotation. After UMAP visualization, 27 clusters corresponding to eight cell types are identified. Differential gene expression among the annotated cell groups is calculated. Next, the intersection of hub genes and differentially expressed genes from single-cell analysis is taken to pinpoint key genes in this study, and their expression is visualized through violin plots.

### 2.9. In Vitro Validation of Potential Key Gene Expression

This study adhered to the standards of the Ethics Committee on Human Experimentation of The First Affiliated Hospital of Sun Yat-sen University, China (2021-334), and the principles of the Declaration of Helsinki (2000). After obtaining informed consent, we obtained samples of synovial membranes from surgical patients. Five OA samples were from five patients who underwent knee arthroplasty, while five control group samples were synovial tissues from patients with unrelated diseases.

Inclusion criteria for OA patients: (1) age ≥50 years; (2) diagnosis of primary knee OA according to the American College of Rheumatology clinical criteria; (3) Kellgren-Lawrence grade III or IV on radiographic examination; (4) scheduled for total knee arthroplasty (TKA) due to end-stage OA; and (5) provision of written informed consent.

Inclusion criteria for control subjects: (1) age ≥50 years; (2) no history of OA, rheumatoid arthritis, or other inflammatory arthropathies; (3) synovial tissue obtained during arthroscopic surgery or open procedures for non-OA conditions (e.g., meniscal injury, ligament reconstruction, or trauma without joint degeneration); and (4) provision of written informed consent.

Exclusion criteria for both groups: (1) history of intra-articular corticosteroid or hyaluronic acid injection within 6 months prior to surgery; (2) previous knee surgery on the index joint; (3) diagnosis of inflammatory arthritis, septic arthritis, or crystal arthropathy; (4) systemic diseases affecting bone metabolism (e.g., osteoporosis, Paget’s disease); (5) active malignancy or chemotherapy within the past 5 years; and (6) inability to provide informed consent.

Differential mRNA expression of potential key genes was initially identified using box plots in the GSE82107 and GSE169077 datasets. To further validate these findings, quantitative reverse transcription-polymerase chain reaction (qRT-PCR) was employed to analyze mRNA expression levels of key genes in synovial tissues from OA patients and non-OA controls. Total RNA was extracted from synovial tissues using TRIzol reagent (Invitrogen, Carlsbad, CA, USA; 15596026) according to the manufacturer’s protocol. RNA concentration and purity were assessed using a NanoDrop 2000 spectrophotometer (Thermo Fisher Scientific, Waltham, MA, USA), with samples having A260/A280 ratios between 1.8 and 2.1 considered acceptable. Reverse transcription was performed using the PrimeScript RT Reagent Kit (Takara Bio, Kusatsu, Shiga, Japan; RR037A) with the following conditions: 37 °C for 15 min, followed by 85 °C for 5 s, using 1 μg of total RNA in a 20 μL reaction volume. Quantitative PCR was performed using the ROX-containing SYBR Green Pro Taq HS qPCR Master Mix II (Accurate Biology, Changsha, Hunan, China) on an ABI ViiA 7Dx Real-Time PCR System (Applied Biosystems, Foster City, CA, USA). The 20 μL reaction mixture contained 10 μL of 2× SYBR Green Master Mix, 0.8 μL of each primer (10 μM), 2 μL of cDNA template, and 6.4 μL of nuclease-free water. The thermal cycling conditions were: initial denaturation at 95 °C for 30 s, followed by 40 cycles of 95 °C for 5 s and 60 °C for 30 s. A melting curve analysis was performed after each run to confirm amplification specificity. GAPDH was used as the internal reference gene. Relative gene expression was calculated using the 2^(−ΔΔCt) method. Primer sequences are listed in Supplementary Table S1.

For protein-level validation [36], total protein was extracted from synovial tissues using RIPA lysis buffer (Beyotime Biotechnology, Shanghai, China; P0013B, China) supplemented with 1% protease inhibitor cocktail (Beyotime, P1005) and 1% phosphatase inhibitor cocktail (Beyotime, P1081). Tissues were homogenized on ice using a tissue grinder, followed by centrifugation at 12,000× *g* for 15 min at 4 °C. Protein concentrations were quantified using the BCA assay kit (Beyotime, P0012) according to the manufacturer’s instructions to ensure equal loading. A total of 30 μg of protein per sample was loaded onto 8–12% gradient SDS-PAGE gels (depending on the molecular weight of the target protein: 8% for EGR1 and HDAC4; 10% for PFKFB3 and PDK4; 12% for MMP13 and GAPDH) and separated by electrophoresis at 80 V for 30 min, followed by 120 V for 60 min. Proteins were then transferred to 0.45 μm PVDF membranes (Millipore, Billerica, MA, USA) at 250 mA for 90 min at 4 °C. After transfer, membranes were blocked with 5% non-fat dry milk in TBST (Tris-buffered saline containing 0.1% Tween-20) for 1 h at room temperature, followed by overnight incubation at 4 °C with the following primary antibodies diluted in 5% BSA/TBST: EGR1 (1:1000, Proteintech, Cat No. 55117-1-AP), PFKFB3 (1:1000, Proteintech, Cat No. 13763-1-AP), HDAC4 (1:800, Proteintech, Cat No. 17449-1-AP), MMP13 (1:1000, Proteintech, Cat No. 18165-1-AP), PDK4 (1:1000, Proteintech, Cat No. 12949-1-AP), and GAPDH (1:5000, Proteintech, Cat No. 60004-1-Ig). After washing three times with TBST (10 min each), membranes were incubated with HRP-conjugated secondary antibodies (goat anti-rabbit IgG, 1:5000, Proteintech, Cat No. SA00001-2; goat anti-mouse IgG, 1:5000, Proteintech, Cat No. SA00001-1) for 1 h at room temperature. Chemiluminescent detection was performed using an ECL kit (Millipore Billerica, MA, USA; WBKLS0500). Protein bands were visualized using a ChemiDoc XRS+ imaging system (Bio-Rad Laboratories, Hercules, CA, USA; USA), and densitometric quantification was performed using Image Lab software (version 6.0, Bio-Rad). GAPDH served as a loading control to ensure data reliability. Relative protein expression was calculated as the ratio of target protein band intensity to GAPDH band intensity.

Immunofluorescence staining provided a direct visualization of target protein distribution and expression. Synovial cells were stained following a previously described protocol [37]. Primary antibodies used in this study included those against EGR1, PFKFB3, HDAC4, MMP13, and PDK4. Samples were subsequently incubated with corresponding secondary antibodies and counterstained with 4′,6-diamidino-2-phenylindole (DAPI). Images were acquired at various magnifications using a confocal laser microscope (Olympus, Tokyo, Japan). Fluorescence intensity was analyzed using ImageJ (Fiji v2024-05) software.

### 2.10. Verification of the DMM Mice Model

To further analyze the expression of the above-mentioned key genes in OA, we established an OA mouse model through the DMM method and conducted relevant quantitative experiments for verification [38]. The experimental animal procedures were approved by the Animal Research Institute (SYSU-IACUC-2024-002182). Five male, 10-week-old WT C57BL/6J mice were used in the experiment and were all purchased from Jiangsu GemPharmatech, China. All mice underwent the following surgical treatments at 12 weeks of age: DMM surgery on the left knee and sham surgery on the right knee. During the surgery, mice were under flurane (1.5–3%) to ensure pain-free conditions during the surgical process. Postoperatively, mice were placed in a warm, quiet environment for recovery and their behavior and health status were closely monitored. Eight weeks later, the mice were euthanized and the knee joints were collected. For euthanasia, mice were anesthetized with an intraperitoneal injection of an overdose of sodium pentobarbital (150 mg/kg) followed by cervical dislocation to ensure a rapid and humane death. Subsequently, the contents of the knee joint were analyzed using micro-CT, qRT-PCR and IF.

The knee joint tissue was obtained in animal experiments with 4% paraformaldehyde. All joints were scanned using a micro-CT scanner (Zeiss Xradia 510 Versa, Carl Zeiss, Germany) with the following parameters: X-ray source voltage of 80 kV, current of 100 μA, exposure time of 500 ms per projection, 1600 projections over 360° rotation, and a 0.4 mm aluminum filter. The voxel size was set to 10 μm isotropic. Three-dimensional reconstruction and data analysis were carried out using the Multimodal 3D Visualization software (version 1.3, Zeiss, Germany). Based on the results of three-dimensional reconstruction, the sizes of all four supracondylar osteophytes on the medial and lateral sides of the tibia and femur of the knee joint were measured, and the average values were statistically analyzed [39]. At the same time, it should be noted that in the DMM mouse model, the infrapatellar fat pad and synovial membrane are anatomically inseparable due to their small size and intimate adherence. Therefore, the collected knee joint soft tissue samples contained both the IFP and SM as a composite tissue, consistent with the concept of the IFP-SM anatomo-functional unit. The obtained animal experimental tissues were processed and then RT-qPCR measurements were conducted to analyze the RNA expression of key genes. In addition, using a method similar to the previous text, IF analysis was employed to obtain images of the expression of key genes in the synovial tissue of the knee joint of experimental animals.

## 3. Results

### 3.1. Subsection

#### 3.1.1. Technology Roadmap

**Figure 1:**
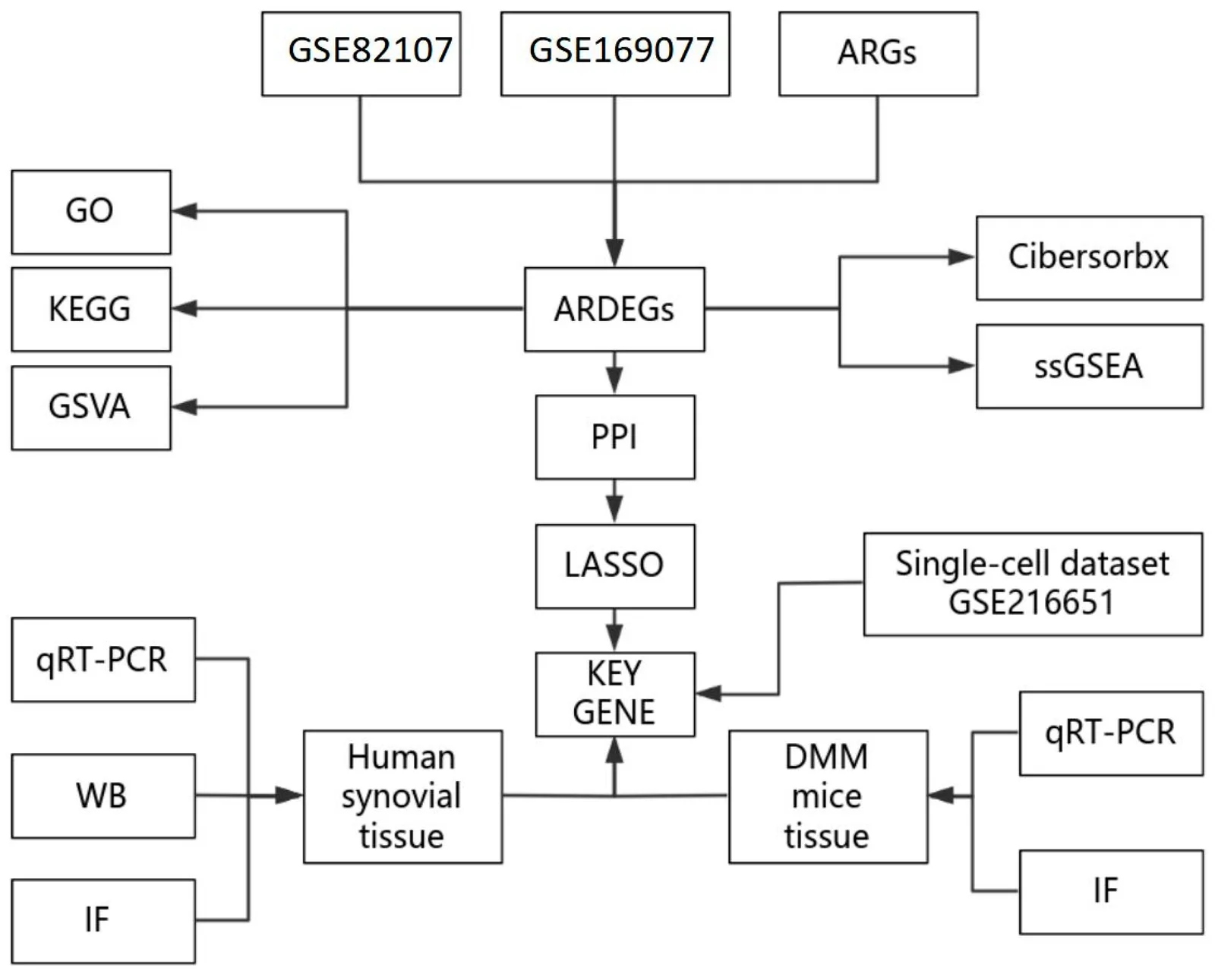
Technical roadmap: Technical roadmap of the study, including the main plans and critical processes.

Figure 1 illustrates the technical roadmap for the study.

#### 3.1.2. Data Collection and Correction

**Figure 2:**
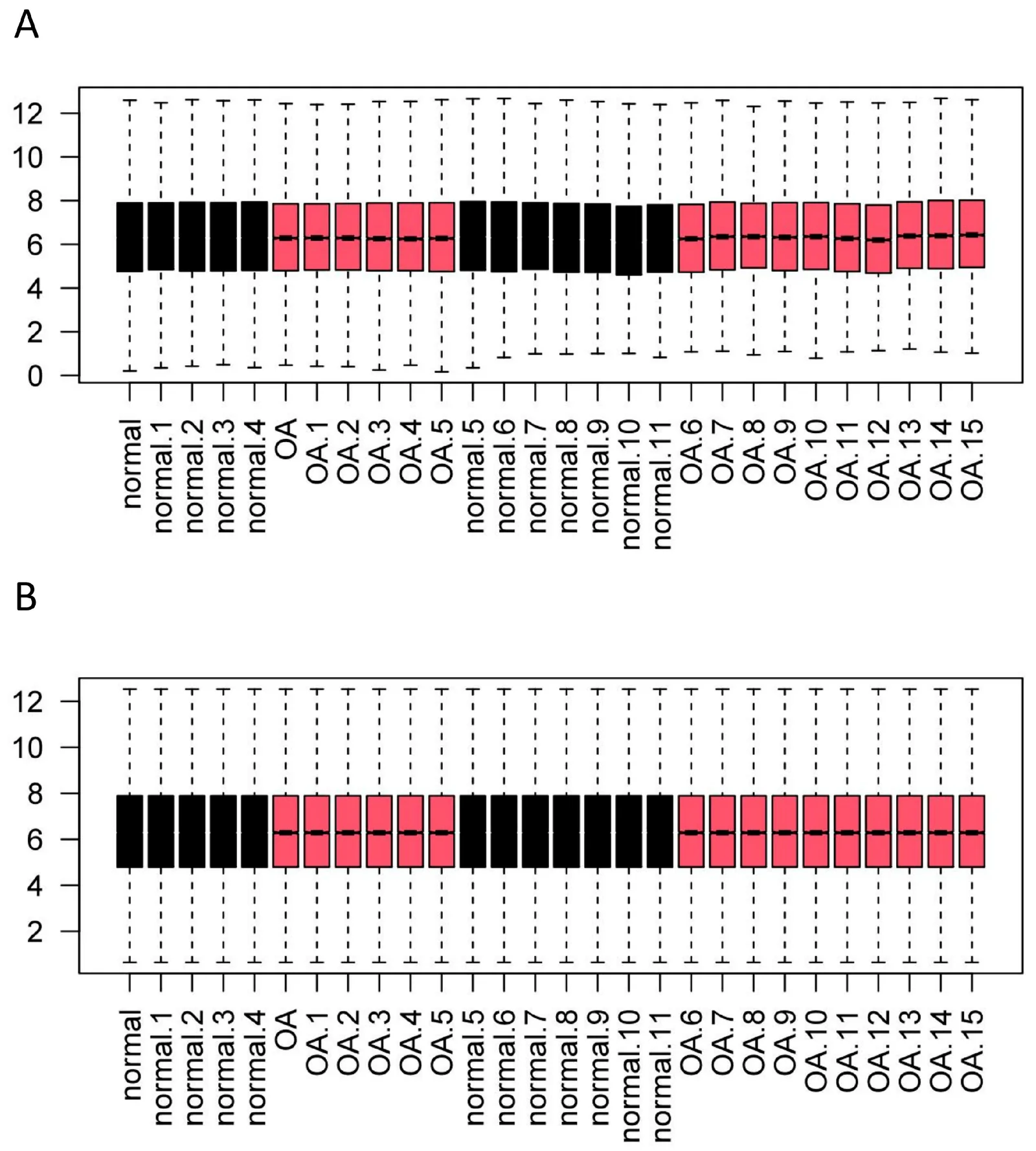
Visualization of the correction analysis before (**A**) and after (**B**) batch-effect correction. It demonstrates the effectiveness of batch effect correction using the ComBat method. Panel A shows boxplots of gene expression distributions across all samples before correction, revealing noticeable inter-batch variability between datasets GSE82107 and GSE169077. Panel B displays post-correction boxplots of gene expression distributions across all samples (*n* = 28). Batch correction was performed using the ComBat method (sva package, R), with dataset origin (GSE82107 vs. GSE169077) as the batch variable and disease status as the preserved biological variable (mod parameter).

The boxplots used to comprehensively assess the datasets before and after batch-effect correction are shown in Figure 2. These visualizations demonstrated significantly reduced batch effects in the merged dataset.

#### 3.1.3. ARDEGs

**Figure 3:**
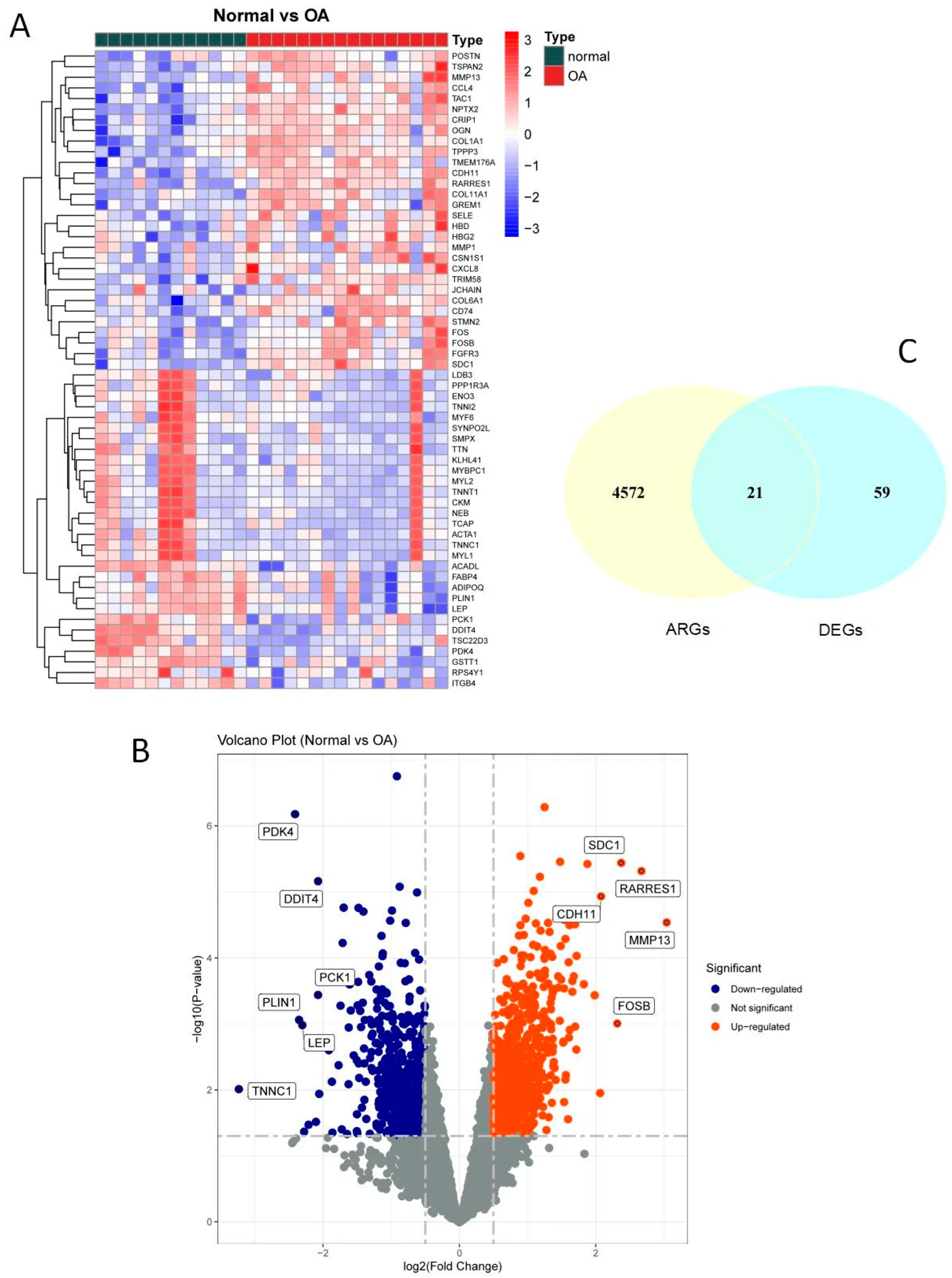
ARDEGs in the OA dataset. (**A**) Heatmap of the top 60 differentially expressed genes with the highest |log_2_FC| values, selected from 80 total DEGs (|log_2_FC| > 1, *p*-adj < 0.05) for clear visualization. (**B**) illustrates the volcano plot distribution, with 188 upregulated genes (red dots, right side) and 147 downregulated genes (blue dots, left side). (**C**) This Venn diagram shows the intersection of 80 differentially expressed genes (in the blue part) and 4592 acetylation-related genes (in the yellow part), resulting in 21 acetylation-related differentially expressed genes (the intersection part).

To identify DEGs, samples from osteoarthritis patients in the combined dataset were designated as the OA group, while samples from healthy individuals were used as the control group. Utilizing the probe annotation platform GPL570 from the GEO database, along with the corresponding R package “hgu133plus2,” we annotated the genes and labeled the expression profiles for both the control and OA groups. Subsequently, we applied the limma package for differential gene expression analysis. The analysis identified 335 DEGs that met the criteria |log2 FC| > 0 and *p*-adj < 0.05, including 188 and 147 upregulated and downregulated genes, respectively, in the OA group compared with the control group. These results are presented in the volcano plot in Figure 3B and the heatmap in Figure 3A. Furthermore, the |log2 FC| > 1 and *p*-adj < 0.05 criteria revealed 80 additional significantly differentially expressed genes (Figure 3C).

The intersection analysis between the 80 significant DEGs identified in the merged dataset and a database of 4592 known ARGs identified 21 ARDEGs: histone deacetylase 4 (*HDAC4*), pyruvate dehydrogenase kinase 4 (*PDK4*), phosphatidylinositol glycan anchor biosynthesis class A (*PIGA*), phosphoenolpyruvate carboxykinase 1 (*PCK1*), beta-1,3-N-acetylgalactosaminyltransferase 1 (*B3GALNT1*), acyl-CoA dehydrogenase long chain (*ACADL*), fucosyltransferase 4 *(FUT4*), early growth response 1 (*EGR1*), glycoprotein hormones, alpha polypeptide (*CGA*), twist family bHLH transcription factor 1 (*TWIST1*), sulfatase 1 (*SULF1*), cystinosin, lysosomal cystine transporter (*CTNS*), 6-phosphofructo-2-kinase/fructose-2,6-bisphosphatase 3 (*PFKFB3*), tachykinin precursor 1 (*TAC1*), prolyl 3-hydroxylase 2 (*P3H2*), brain abundant, membrane attached signal protein 1 (*BASP1*), serum/glucocorticoid regulated kinase 1 (*SGK1*), collagen-type I alpha 2 chain (*COL1A2*), matrix metallopeptidase 13 (*MMP13*), cysteine-rich protein 1 (*CRIP1*), and tubulin polymerization promoting protein family member 3 (*TPPP3*) (Figure 3C).

#### 3.1.4. PPI Network of ARDEGs

**Figure 4:**
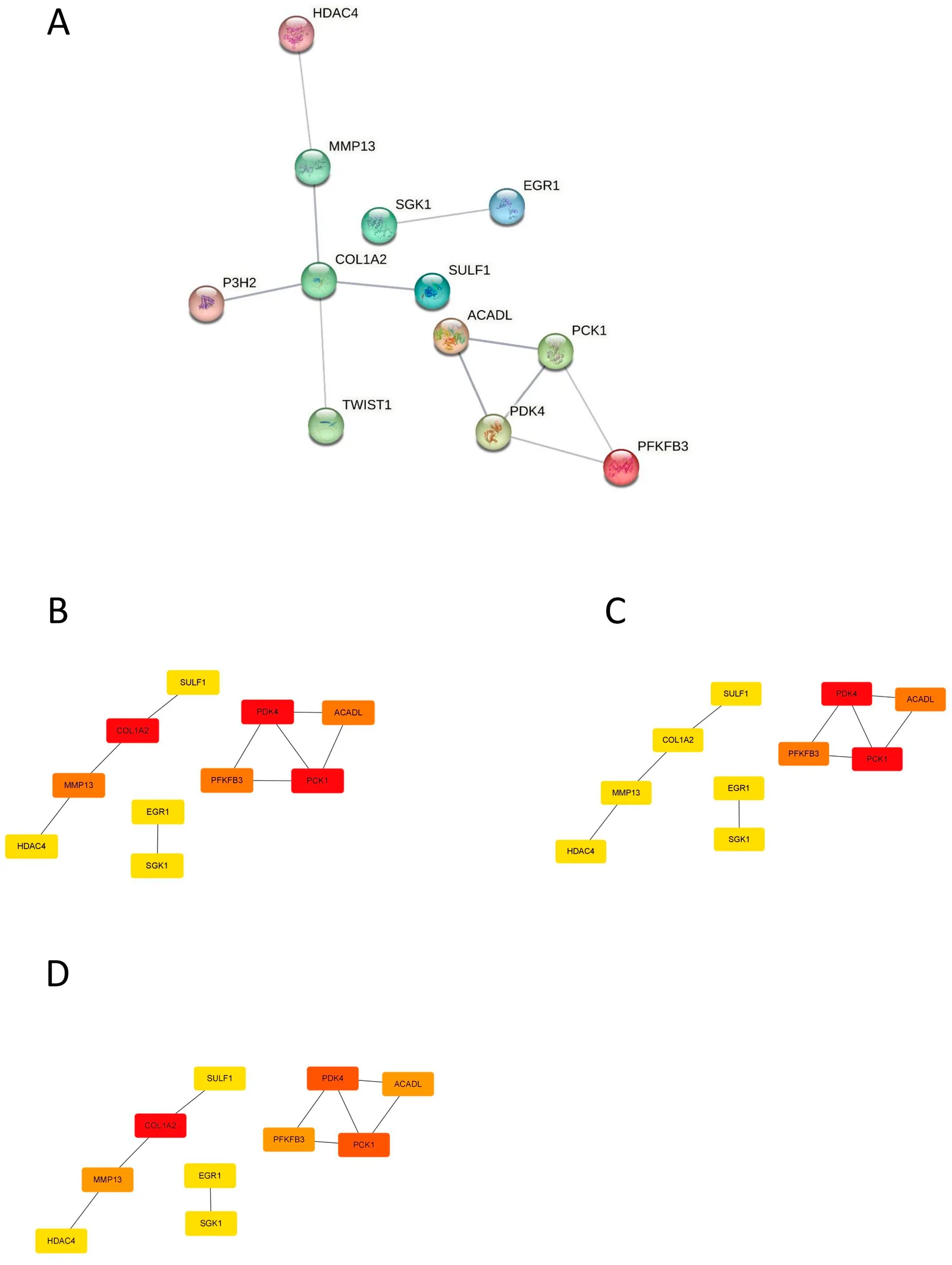
Interaction network of ARDEGs. (**A**) PPI network of the selected ARDEGs. (**B**–**D**) Top 10 genes identified in the PPI network using the MCC, MNC, and degree algorithms, respectively. We use different squares to represent different ratings, where red indicates high and yellow indicates low.

The intricate interactions among genes form complex regulatory networks within cells, and understanding these interactions is crucial for unraveling biological processes and disease mechanisms. We utilized the STRING database to create a PPI network for the 21 ARDEGs, aiming to investigate the interactions among them. Of the 21 ARDEGs, 12 genes (*HDAC4*, *PDK4*, *PCK1*, *ACADL*, *EGR1*, *TWIST1*, *SULF1*, *PFKFB3*, *P3H2*, *SGK1*, *COL1A2*, *MMP13*) exhibited significant protein–protein interactions in the STRING database (minimum interaction score ≥ 0.4) and were retained for PPI network construction. The remaining 9 ARDEGs (*PIGA*, *B3GALNT1*, *FUT4*, *CGA*, *CTNS*, *TAC1*, *BASP1*, *CRIP1*, *TPPP3*) did not meet the interaction threshold and were excluded from network analysis, as their lack of connectivity suggests limited functional coordination with the core acetylation-related gene module in OA (Figure 4A).

To prioritize the most influential genes within the PPI network, we applied three CytoHubba algorithms—MCC, MNC and degree—to score all 12 network nodes. The top 10 genes (*PDK4*, *PCK1*, *COL1A2*, *PFKFB3*, *ACADL*, *MMP13*, *EGR1*, *SULF1*, *SGK1*, *HDAC4*) with the highest combined topological scores were selected as hub genes for subsequent analyses, while *P3H2* and *TWIST1* were excluded due to relatively lower centrality rankings. This step ensures that downstream analyses focus on the most structurally critical nodes within the acetylation-related gene network (Figure 4B–D).

The PPI network reveals two functional modules: Module one is centered on *COL1A2*, connecting ECM-degrading enzyme *MMP13* and transcriptional regulatory factors *EGR1*/*HDAC4*, suggesting that acetylation may affect OA by regulating the balance between matrix synthesis and degradation; Module two is composed of metabolic genes *PDK4*, *PCK1*, *PFKFB3*, and *ACADL*, reflecting the fine regulation of acetylation on energy metabolism. This modular structure supports the hypothesis of ‘structural-metabolic’ dual regulation of acetylation in OA. These identified genes—*PDK4*, *PCK1*, *COL1A2*, *PFKFB3*, *ACADL*, *MMP13*, *EGR1*, *SULF1*, *SGK1*, and *HDAC4*—are essential for network structure and function, and studying these genes may aid in understanding the role of acetylation in OA pathology.

#### 3.1.5. Correlation Analysis of Hub Genes

**Figure 5:**
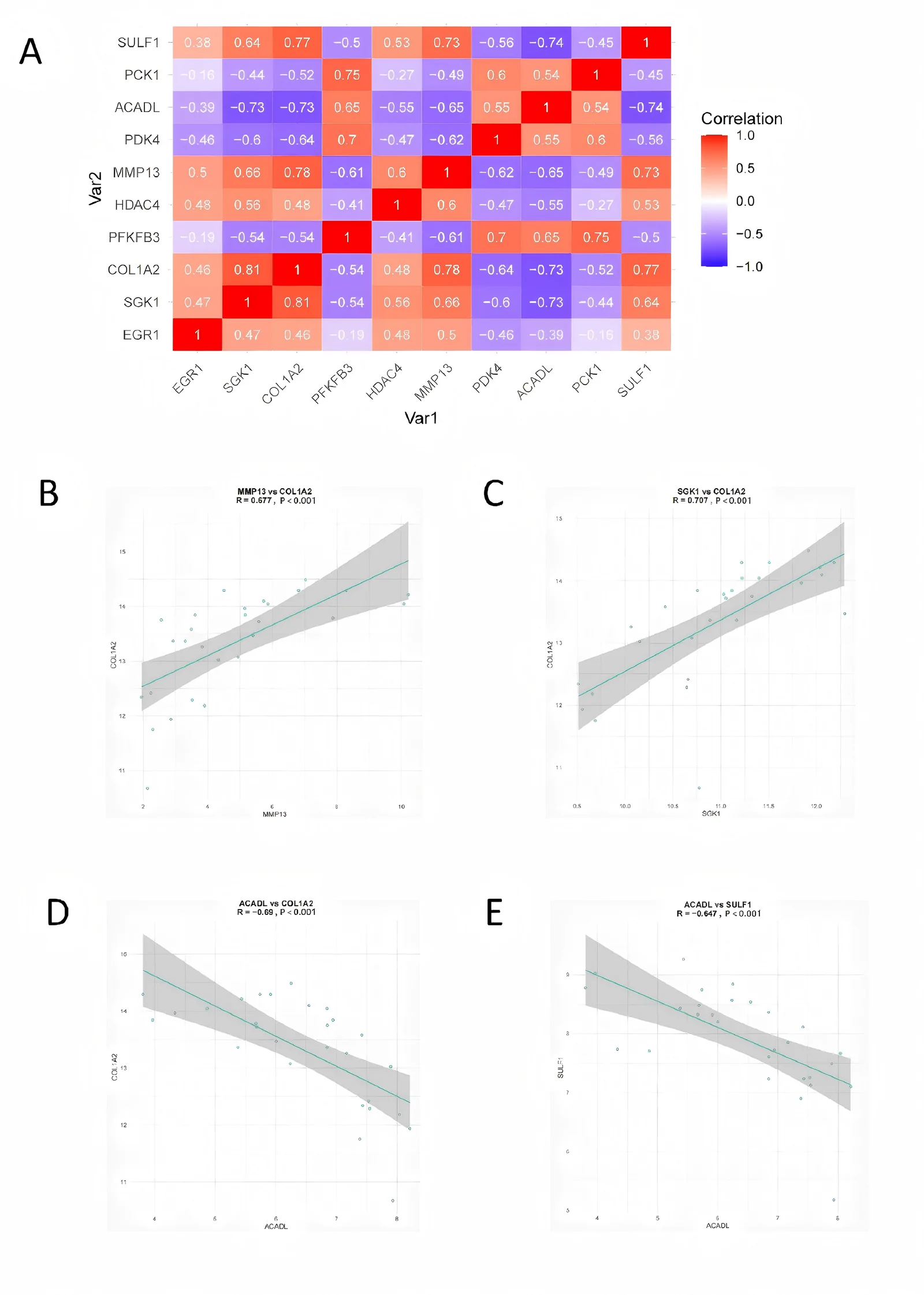
Correlation analysis of hub genes in the combined dataset. (**A**) Correlation heatmap of hub genes in the combined dataset; a higher absolute value of the score indicates a stronger correlation between genes. The Spearman correlation heatmap displays pairwise correlation coefficients among 10 hub genes. (**B**) Correlation between MMP13 and COL1A2. (**C**) Correlation between SGK1 and COL1A2. (**D**) Correlation between ACADL and COL1A2. (**E**) Correlation between ACADL and SULF1. In the correlation scatter plots (**B**–**E**), a correlation coefficient (r) with an absolute value >0.8 indicates a strong correlation; values of 0.5–0.8, 0.3–0.5, and <0.3 suggest moderate, weak, and very weak or no correlation, respectively.

We analyzed the expression matrix of the 10 hub genes in the combined dataset encompassing all samples. Using the Spearman algorithm, we assessed correlations between the expression of these hub genes and visualized the results using a correlation heatmap (Figure 5A). Our results revealed strong associations between the expression of these hub genes. Subsequently, we identified the top two gene pairs with positive and negative correlations from the heatmap and further illustrated their relationships using scatter plots (Figure 5B–E). Specifically, expressions of MMP13 and COL1A2 showed a moderately significant positive correlation, coupling of ECM degradation and synthesis: MMP13-mediated collagen degradation may feedback to activate the reparative expression of COL1A2 (*r* = 0.677, *p* < 0.001; Figure 5B), whereas those of SGK1 and COL1A2 demonstrated a similar moderately significant positive correlation, suggests that SGK1 may promote the maintenance of COL1A2 expression in chondrocytes by regulating ion channels and cell survival. (*r* = 0.707, *p* < 0.001; Figure 5C). In contrast, the expressions of ACADL and COL1A2 exhibited a moderately significant negative correlation, indicating metabolic competition between fatty acid oxidation and collagen synthesis: The reduction of ACADL may redirect metabolic resources to the synthesis of the extracellular matrix. (*r* = −0.69, *p* < 0.001; Figure 5D), whereas those of ACADL and SULF1 exhibited a moderately significant negative correlation. The glycosaminoglycan modification controlled by SULF1 and the fatty acid oxidation process may have a mutually regulating relationship in terms of metabolism and structure.(*r* = −0.647, *p* < 0.001; Figure 5E).

#### 3.1.6. LASSO Regression Analysis

**Figure 6:**
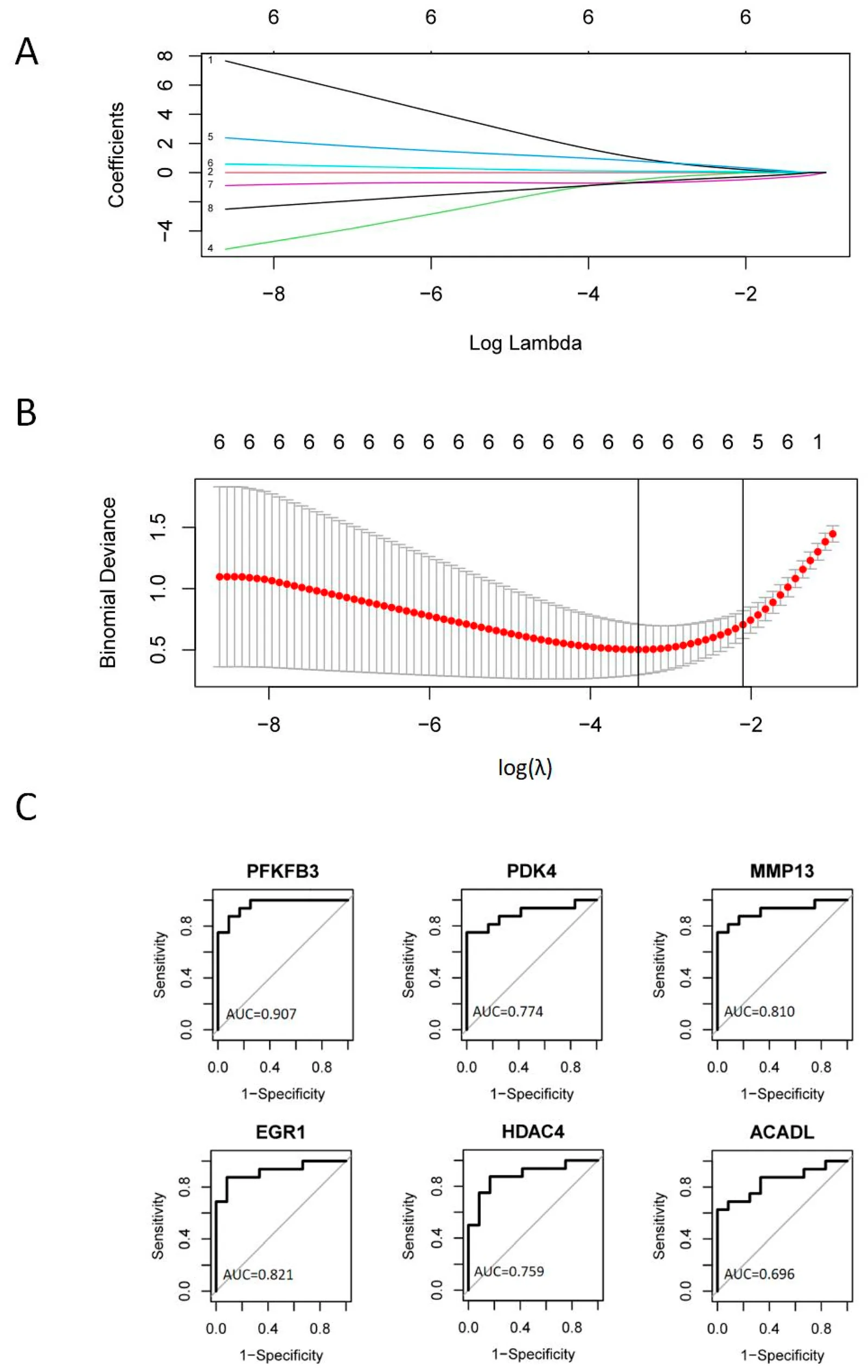
Results of the LASSO analysis. (**A**) Coefficient paths showing trajectories of regression coefficients for hub genes as the penalty parameter λ increases. Each curve represents one gene; the y-axis shows coefficient values, and the x-axis shows log(*λ*). As λ increases, coefficients of some genes are shrunk to zero, achieving variable selection. (**B**) In the cross-validation curve, red dots indicate binomial deviance at different log(*λ*) values; gray error bars represent standard errors. The left vertical dashed line indicates the optimal λ value (lambda.min, log(*λ*) ≈ −3.5), where the model achieves minimum cross-validation error and 6 genes retain non-zero coefficients (*EGR1*, *PFKFB3*, *HDAC4*, *MMP13*, *PDK4*, *ACADL*). This model was selected as the final diagnostic signature. (**C**) Expression differences of key genes between patients with OA and those without the condition. ROC curves and AUC values for the 6 LASSO-selected hub genes in the combined dataset (n = 28; 16 OA, 12 Control). AUCs were calculated using the pROC package (R). Dashed diagonal indicates the reference line (AUC = 0.5).

We identified key hub genes through LASSO regression analysis and assessed their potential application in the diagnosis of OA using ROC curves. As shown in Figure 6, as the penalty increased, the regression coefficients of the four genes decreased to zero, indicating that these genes were not critical predictive factors in the model (Figure 6A,B). The remaining six genes were identified as potential hub genes, and their regression coefficients were not significantly equal to zero (*p* < 0.05), suggesting their significant role in differentiating OA from normal samples. These genes included *EGR1*, *PFKFB3*, *HDAC4*, *MMP13*, *PDK4* and *ACADL*.

LASSO analysis refined the 10 hub genes to 6 key genes (*EGR1*, *PFKFB3*, *HDAC4*, *MMP13*, *PDK4*, *ACADL*) with non-zero coefficients, achieving AUC values of 0.688–0.907 in ROC analysis. These 6 genes represent a parsimonious yet robust signature for OA diagnosis (Figure 6C).

**Figure 7:**
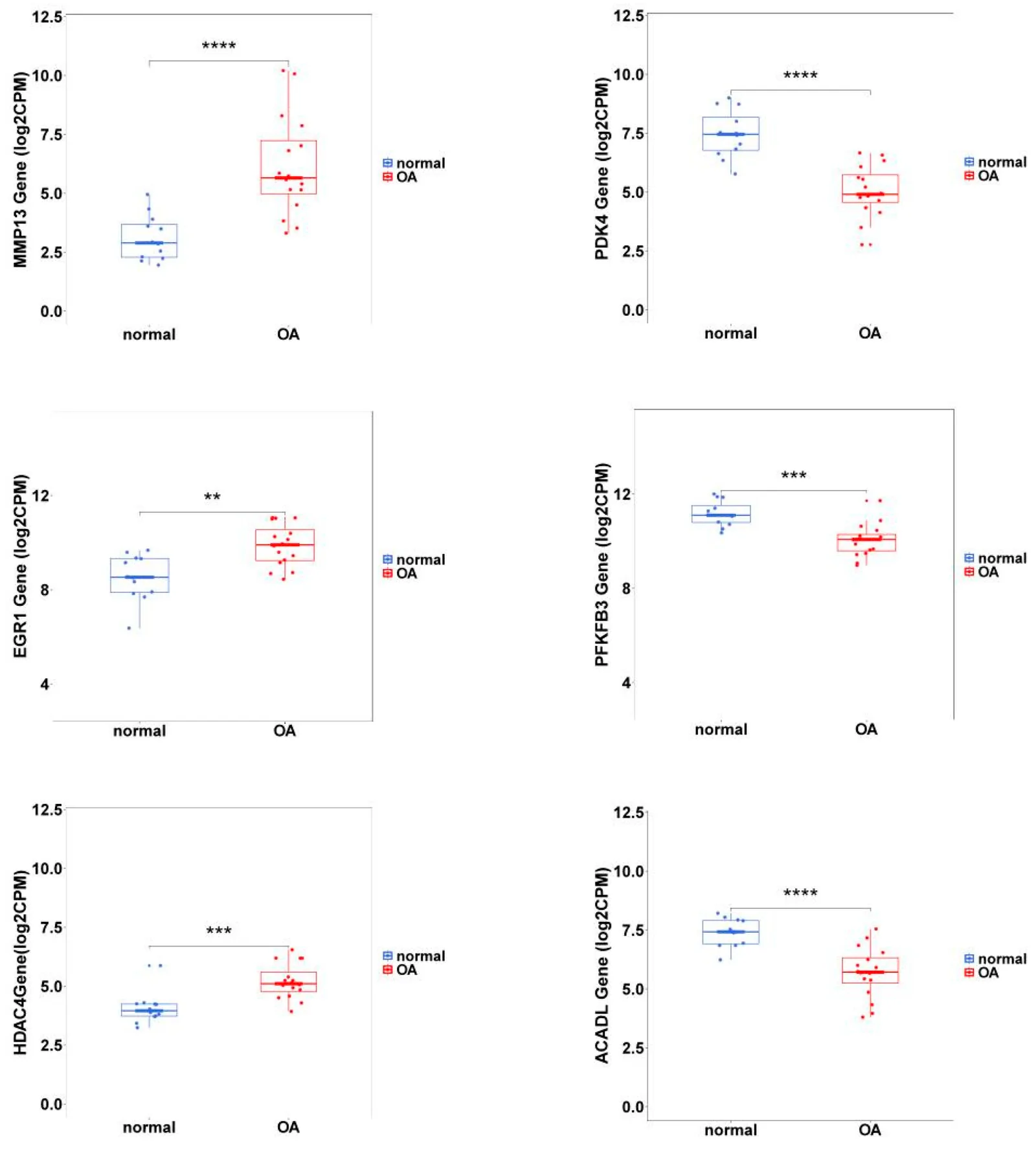
Boxplots display log2CPM expression levels of 6 hub genes across normal (blue, *n* = 12) and OA (red, *n* = 16) samples. Significantly upregulated genes in OA include MMP13, *EGR1*, and *HDAC4*. Significantly downregulated genes include *PDK4*, *PFKFB3*, and *ACADL*. Asterisks indicate statistical significance: * *p* < 0.05, ** *p* < 0.01, *** *p* < 0.001 and **** *p* < 0.0001.

The boxplots comparing the gene expressions between OA and normal samples revealed significantly higher expressions of *MMP13*, *EGR1* and *HDAC4* in OA samples compared with normal samples (*p* < 0.05). Conversely, *PDK4*, *PFKFB3* and *ACADL* exhibited lower expressions in the OA samples. The differential expression of these genes may be associated with OA pathogenesis (Figure 7).

#### 3.1.7. Immune Infiltration Analysis

**Figure 8:**
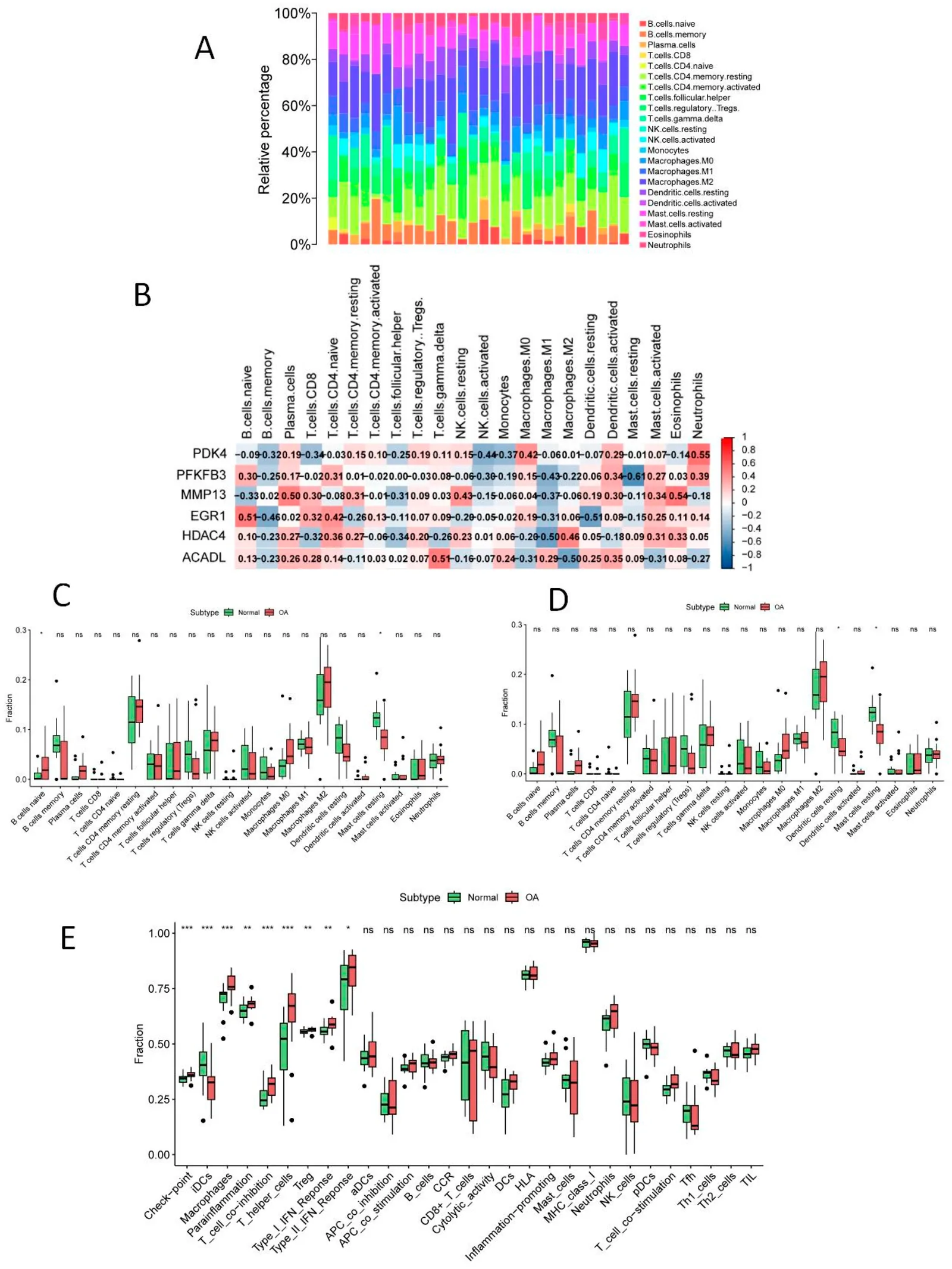
Results using the CIBERSORTx immune infiltration assay. (**A**) Stacked bar chart of immune cell infiltration in samples from the combined datasets as determined using the CIBERSORTx algorithm. (**B**) The heatmap depicts the relationships regarding immune cell infiltration levels and hub gene expression within the integrated dataset. (**C**) presents t-test comparisons of CIBERSORTx-estimated cell proportions, showing nominal (unadjusted) differences with a decreasing trend in resting mast cells (*p* = 0.010) and an increasing trend in naive B cells (*p* = 0.041) in OA; none remained significant after Benjamini–Hochberg FDR correction. (**D**) Rank-sum test boxplot for grouped CIBERSORTx results. The abundance of resting dendritic cells and resting mast cells decreased in OA. (**E**) Boxplot for grouped ssGSEA results displays ssGSEA functional pathway scores, revealing significant upregulation of interferon responses, macrophage function, and T cell co-inhibition pathways. (Black dots in panels C–E indicate individual outliers; “ns” denotes non-significant differences.) * *p* < 0.05, ** *p* < 0.01, *** *p* < 0.001.

To discern the variations in immune cell infiltration between the OA and controls, we applied the CIBERSORTx algorithm to quantify the abundance of 22 distinct immune cell types. The stacked bar chart delineating the relative proportions of immune cell infiltration revealed disparities between the groups across 22 cell types (Figure 8A).

Correlation analysis revealed selective associations between the 6 LASSO-selected key genes and specific immune cell types: *PDK4* positively correlated with neutrophils (*r* = 0.55), raising the hypothesis that *PDK4* metabolic status might be co-regulated with neutrophil abundance; *PFKFB3* negatively correlated with activated mast cells (*r* = −0.61), consistent with a speculative inverse association between glycolytic flux and mast cell activation; and *MMP13* positively correlated with eosinophils (*r* = 0.54), suggesting potentially pathological synergy between ECM degradation and granule protein release (Figure 8B).

The analysis results showed that only two types of immune cell types exhibited nominally significant changes in OA by Student’s *t*-test (Figure 8C): the abundance of naive B cells (B.cells.naive) increased in OA (*p* = 0.041), suggesting that there might be B-cell-mediated humoral immune activation or autoantibody production in the OA synovium; the abundance of resting mast cells (Mast.cells.resting) decreased in OA (*p* = 0.010), while the proportion of activated mast cells (Mast.cells.activated) showed no significant difference. The non-parametric Wilcoxon rank sum test was used to verify the robustness of the t-test results (Figure 8D). The comparison between the normal control and OA patients showed that only two cell types presented consistent differences: the abundance of resting dendritic cells (Dendritic.cells.resting) decreased in OA (*p* = 0.049), suggesting a similar depletion of the dendritic cell pool; similarly, the abundance of resting mast cells (Mast.cells.resting) decreased in OA (*p* = 0.015). The pattern of mast cell abundance changes is different from the classical resting-to-activated transformation hypothesis, and is more likely to reflect the progressive depletion of the mast cell pool in the OA synovium (Figure 8C,D)—that is, continuous chronic inflammation leads to impaired recruitment, reduced survival, or phenotypic drift of mast cells, rather than a simple functional state transition. As a key effector cell of joint inflammation, the depletion of mast cells may be involved in the sensitization of OA pain and the fibrotic process of the synovium (Figure 8C,D). Importantly, after applying the Benjamini–Hochberg (BH) correction for multiple testing across the 22 immune cell types, none of these differences remained statistically significant (all FDR-adjusted *p* > 0.05; smallest adjusted *p* = 0.228). Therefore, although these trends may still hint at B-cell-mediated humoral immune activation and mast cell pool depletion in the OA synovium, the immune infiltration findings should be interpreted with caution as exploratory observations.

ssGSEA revealed significant enrichment score disparities for 28 immune cell populations between normal and OA synovial samples. In contrast to the limited differences observed in the CIBERSORTx cell type analysis, ssGSEA revealed that multiple immune pathways in OA patients were significantly activated: the I/II type interferon response, macrophage function, checkpoint molecules, T cell co-inhibition, and helper T cell pathways were all upregulated (*p* < 0.05) (Figure 8E). This pattern of “stable cell numbers but highly activated functions” indicates a chronically activated inflammatory state in the OA synovium, further revealing how differentially expressed genes regulate the OA immune microenvironment and the potential regulatory role of acetylation modifications.

#### 3.1.8. Enrichment Analysis

**Figure 9:**
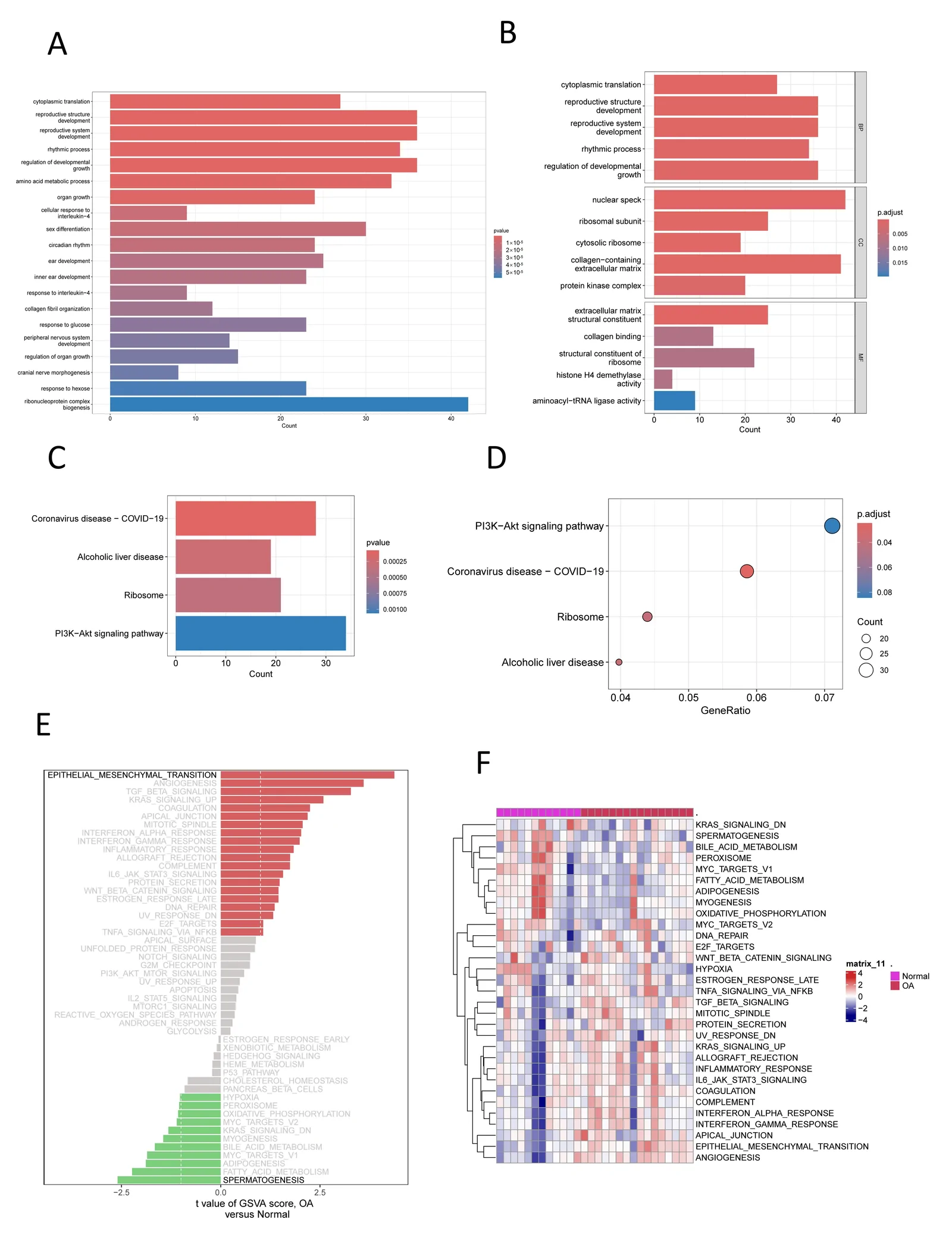
Enrichment Analysis Results. (**A**,**B**) GO enrichment analysis of 80 DEGs (biological process, cellular component, molecular function). Enrichment significance was determined by clusterProfiler (R) with adjusted *p*-value < 0.05. (**C**,**D**) KEGG pathway enrichment analysis. (**E**,**F**) GSVA hallmark pathway scores across individual samples (*n* = 28), stratified by disease status.

GO annotation of DEGs displayedthe top 10 significant terms across BP, CC, and MF. BP analysis highlighted “cytoplasmic translation,” “reproductive structure development,” and “rhythmic process,” indicating dysregulated protein synthesis and developmental program reactivation in OA; notably, “amino acid metabolic process” and “organ growth” enrichment reflected metabolic reprogramming-tissue remodeling synergy. CC analysis showed dominance of “nuclear speck” (enriched in pre-mRNA splicing factors), “ribosomal subunit,” and “cytosolic ribosome,” pointing to post-transcriptional dysregulation; “collagen-containing extracellular matrix” enrichment possibly corresponded to structural functions of MMP13. MF analysis emphasized “ECM structural constituent” and “collagen binding” activities, reinforcing ECM homeostasis imbalance. These GO entries collectively form a pathological chain from transcription to translation to ECM output, providing new pathogenic hypotheses for understanding the multi-level regulation of acetylation in OA (Figure 9A,B).

The bar charts and bubble charts present the top 4 significant pathways. The relationships between these pathways and OA, as well as acetylation, might be as follows. The PI3K-Akt signaling pathway ranks first. This pathway integrates cell survival, metabolism, and immune signals and is regulated by acetylation modification (the deacetylation of Akt affects its kinase activity). The enrichment of the coronavirus disease-COVID-19 pathway may stem from the shared inflammatoryosome activation and cytokine storm mechanism between OA and viral inflammation rather than a direct pathological association. The ribosome pathway corresponds to the translation entries of GO, suggesting an overall dysregulation of the protein synthesis machinery. The enrichment of the alcoholic liver disease pathway reflects the cross-disease commonality of metabolic stress and oxidative damage mechanisms (Figure 9C,D; Supplementary Tables S2–S4).

The results of GSVA demonstrated that the upregulated pathway ranked highest was “Epithelial-Mesenchymal Transition” (EMT), which shares molecular programs with chondrocyte hypertrophic differentiation (e.g., Snail/Slug transcription factors, N-cadherin expression), suggesting that OA chondrocytes acquire migratory and proliferative phenotypes. Activation of “Angiogenesis” and “TGF-β Signaling” corresponds to synovial pannus formation and fibrosis, respectively; whereas “KRAS Signaling Up” may reflect inflammation-driven aberrant activation of the RAS pathway. Among downregulated pathways, “Myogenesis,” “Adipogenesis,” and “Fatty Acid Metabolism” were significantly suppressed, consistent with the downregulation of hub genes *PDK4*, *PFKFB3*, *ACADL*, and *PCK1*, corroborating metabolic reprogramming in OA synovium and cartilage—a shift from fatty acid oxidation toward glycolysis. Heatmap visualization of sample-level pathway activity heterogeneity revealed distinct stratification of activation intensity among OA samples (Figure 9E,F).

#### 3.1.9. Verification of Single-Cell RNA Sequencing Data

**Figure 10:**
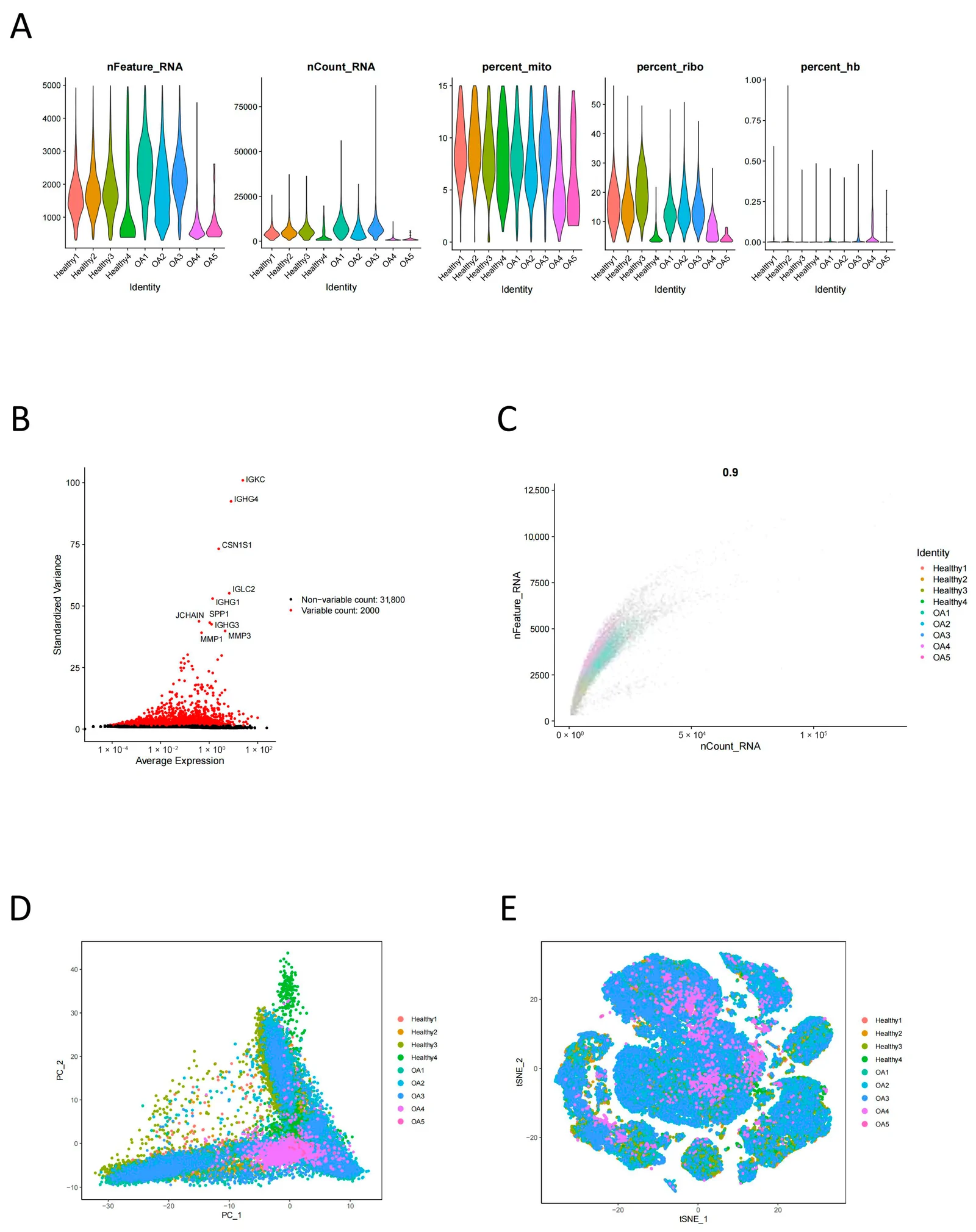
Please add figure caption here.

To explore the expression patterns of ARGs in OA patients, we obtained the single-cell sequencing dataset GSE216651 and conducted analyses. Data were filtered based on the number of gene characteristics, the number of gene counts, the percentage of mitochondrial gene expression, the percentage of ribosomal gene expression, and the percentage of hemoglobin gene expression (Figure 10A). We then extracted the 33,800 genes for PCA analysis (Figure 10B) and used the R package harmony to remove batch effects. A high correlation was observed in the nCount vs. nFeature scatter plot (Figure 10C), indicating effective preprocessing. Clear cell-cluster distributions in the single-cell dimensionality reduction plot (Figure 10D,E) further confirmed effective batch-effect removal.

**Figure 11:**
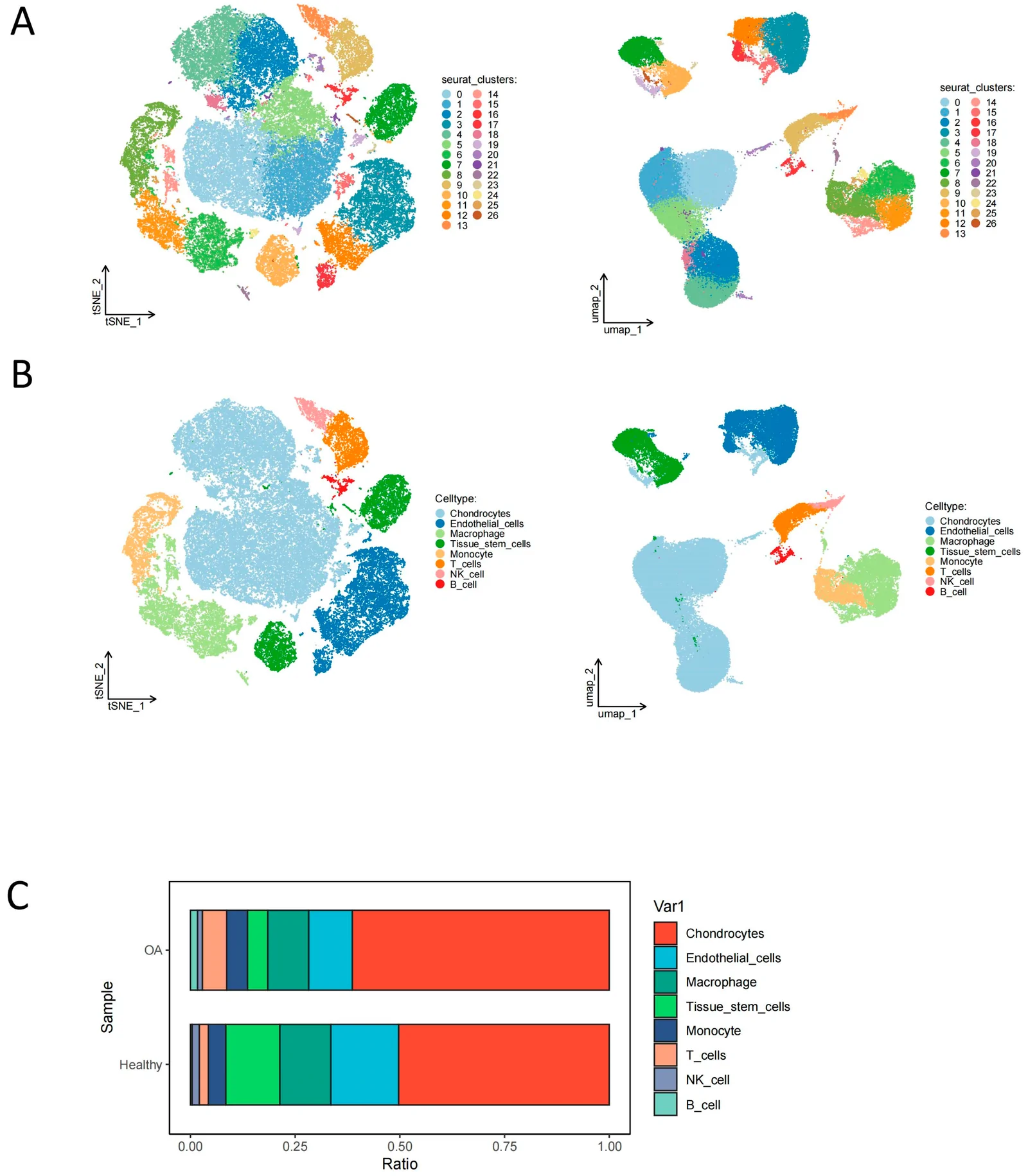
Cell Type Annotation and Key Gene Expression. (**A**) Unsupervised clustering results. t-SNE (left) and UMAP (right) display the 27 Seurat clusters (seurat_clusters 0–26), with batch effects controllable after Harmony integration, and the cell population structure is clear. (**B**) Cell type annotation. Based on the automatic annotation by SingleR combined with manual correction using literature markers, 27 clusters were classified into 8 cell types: chondrocytes (Chondrocytes, light blue, the largest group), endothelial cells (Endothelial_cells, dark blue), macrophages (Macrophage, green), tissue stem cells (Tissue_stem_cells, yellow-green), monocytes (Monocyte, orange), T cells (T_cells, red), NK cells (NK_cell, pink), and B cells (B_cell, deep red). The UMAP spatial distribution shows the regional separation of chondrocytes from immune cells. (**C**) Comparison of sample composition. The stacked bar chart shows the proportion of cell types in normal and OA samples. Both groups are dominated by chondrocytes (~50%), but in the OA samples, the proportion of macrophages/monocytes is relatively higher, while the proportion of T cells is lower, reflecting the inflammatory infiltration characteristics of the OA synovium/Cartilage.

**Figure 12:**
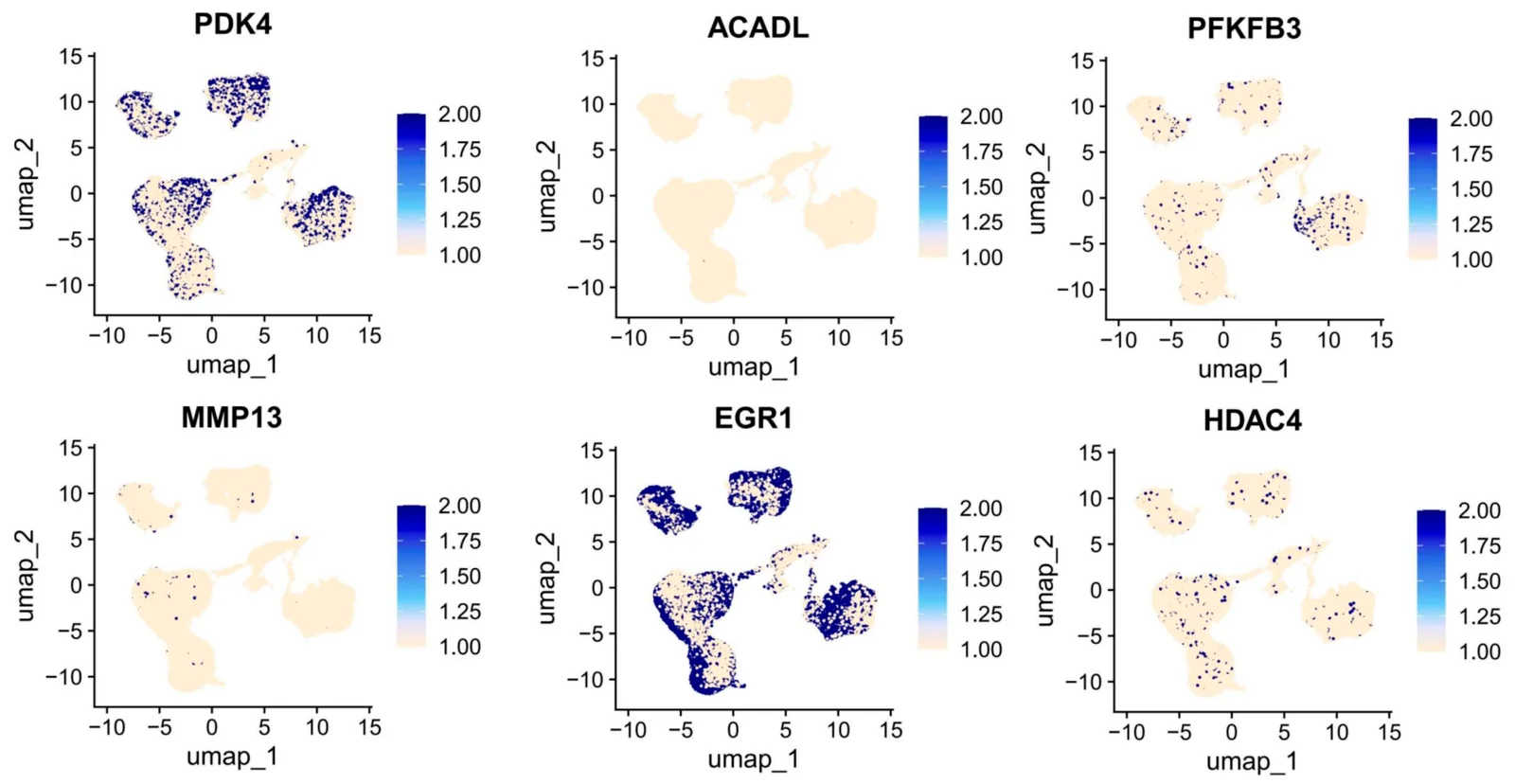
The UMAP visualization shows the expression intensity distribution of 6 key genes in the integrated dataset of all samples. Expression patterns represent combined data from 4 healthy donors and 5 OA patients after Harmony batch correction. The blue gradient represents low expression (1–2) and high expression (>2).

In the processed GSE216651 dataset, we used the singleR method in R to annotate 27 cell clusters (Figure 11A). Based on cell-type-specific markers, the 27 clusters were classified into 8 cell types: Chondrocytes, Endothelial cells, Macrophage, Tissue stem cells, Monocyte, T cells, NK cells, and B cells (Figure 11B). A stacked bar plot was generated to show the distribution of these 8 cell types across different groups (Figure 11C). Finally, UMAP plots were used to validate the expression patterns of 6 key genes in the different cell clusters (Figure 12).

**Figure 13:**
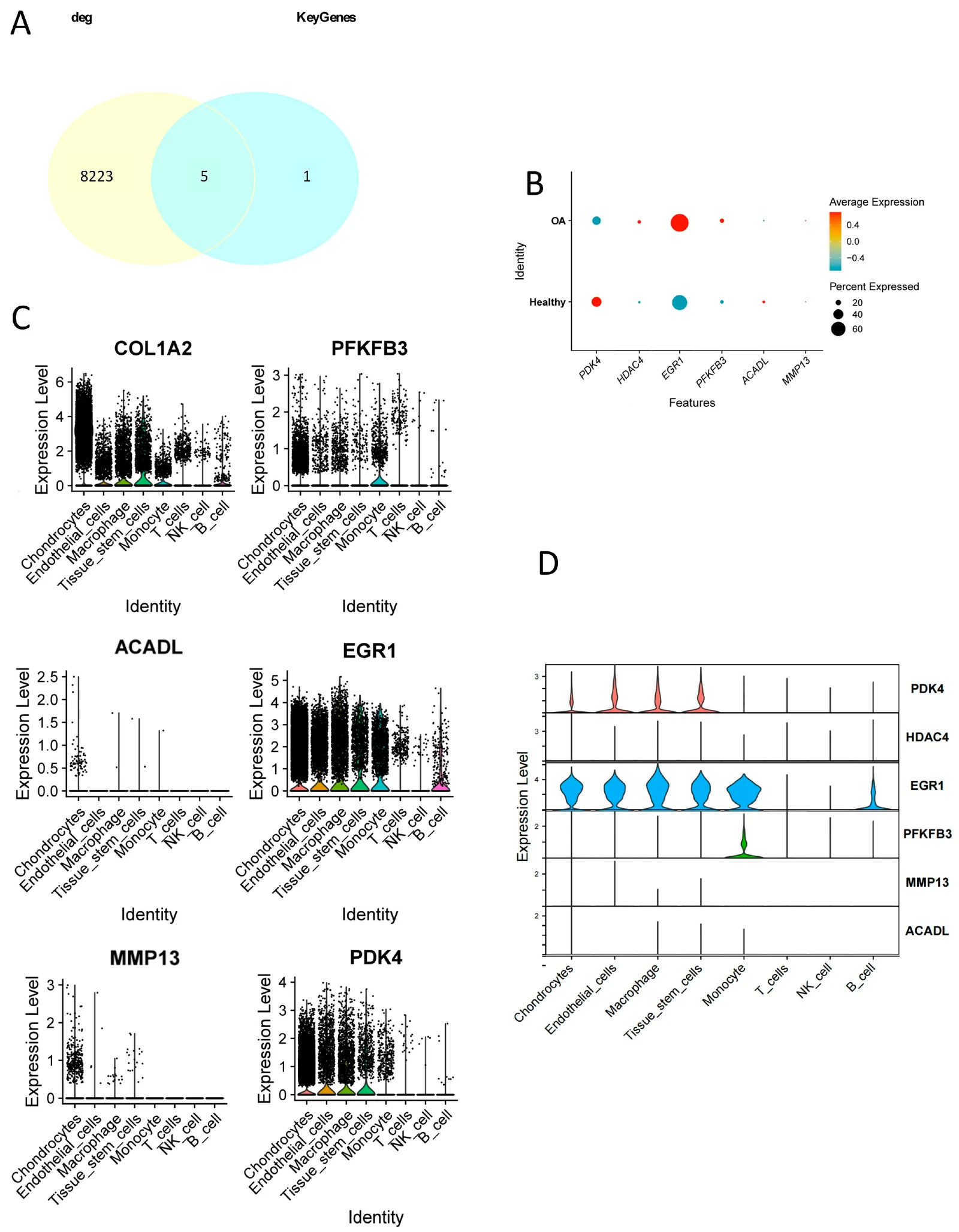
Identification and Analysis of Key Genes. (**A**) The Venn diagram shows that the intersection of the 8228 differentially expressed genes identified by single-cell analysis and the 6 hub genes is 5 genes (*EGR1*, *MMP13*, *HDAC4*, *PFKFB3*, *PDK4*), while *ACADL* does not overlap. (**B**) The bubble chart shows the average expression levels (color) and expression ratios (size) of 6 genes in the two groups. (**C**,**D**): Violin plots of key gene expression across cell types display expression distribution by cell type without disease-state stratification.

We intersected the differentially expressed genes from the GSE216651 dataset with the previously identified 6 central genes. Among these 6 genes, 5 showed significant intergroup differences between groups in the single-cell analysis data (Figure 13A). Among them, no significant intergroup differences were observed for *ACADL* in the single-cell analysis. Considering other data, the possible reason is the combined limitation of gene expression characteristics and the sensitivity of the detection technology. In the single-cell results of the GSE216651 dataset, *ACADL* has extremely low basal expression in chondrocytes and macrophages (<1 UMI per cell), approaching the lower limit of detection for single-cell sequencing.

A bubble plot was used to compare the expression differences of these 6 key genes between the OA group and normal controls (Figure 13B). To determine the cellular localization of the 6 hub genes, violin plots were generated to illustrate the expression of these 6 key genes across the 8 cell types. Specifically, *EGR1* is highly enriched in chondrocytes; *PFKFB3* is predominantly expressed in macrophages (Figure 13C,D).

#### 3.1.10. In Vitro Validation

**Figure 14:**
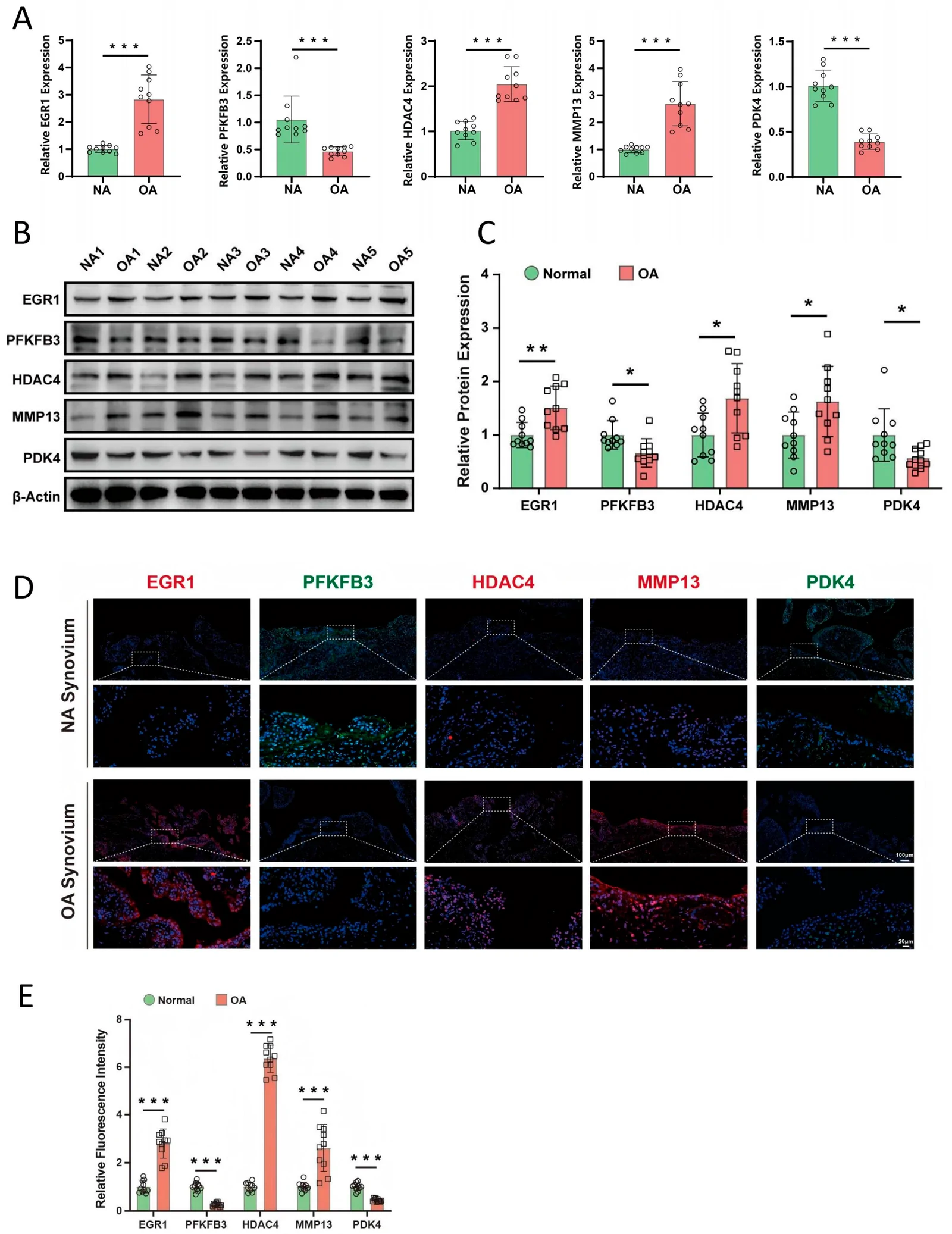
Validation of Acetylation-Related Key Genes. (**A**) qRT-PCR analysis revealed significantly upregulated mRNA levels of *EGR1*, *HDAC4*, and *MMP13*, and downregulated mRNA levels of *PFKFB3* and *PDK4* in OA tissues compared with controls. (**B**,**C**) Western blot analysis demonstrated higher relative protein expression levels of *EGR1*, *HDAC4*, and *MMP13*, and lower levels of *PFKFB3* and *PDK4* in OA tissues than in controls. (**D**,**E**) Immunofluorescence staining showed the same results as qRT-PCR and WB. Asterisks indicate statistical significance: * *p* < 0.05, ** *p* < 0.01, and *** *p* < 0.001.

qRT-PCR analysis confirmed the bioinformatics findings, revealing differences in mRNA expression levels of five key genes (*EGR1*, *PFKFB3*, *HDAC4*, *MMP13*, and *PDK4*) between synovial tissues of OA patients and controls (*p* < 0.001). Specifically, *EGR1*, *HDAC4*, and *MMP13* were upregulated, while *PFKFB3* and *PDK4* were downregulated in OA tissues (Figure 14A). Calculated using the *2^−^ΔΔCt* method and expressed as Mean ± SEM, these results suggest a potential role of these genes in OA pathogenesis.

Western blotting further validated differential protein expression (Figure 14B,C). Consistent results showed that *EGR1*, *HDAC4*, and *MMP13* proteins were upregulated in OA tissues compared with controls (*p* < 0.05), with *MMP13* exhibiting the most significant increase (*p* < 0.01). In contrast, *PFKFB3* and *PDK4* protein levels were significantly downregulated (*p* < 0.05).

Immunofluorescence staining provided a direct visualization of target protein localization and expression differences (Figure 14D,E). In OA tissues, *EGR1*, *HDAC4*, and *MMP13* proteins were upregulated in OA tissues compared with controls, while *PFKFB3* and *PDK4* protein levels were significantly downregulated (*p* < 0.001).

#### 3.1.11. The Expression of Key Genes Also Yielded Similar Results in the Knee Joint Tissue of the OA Model in DMM Mice

**Figure 15:**
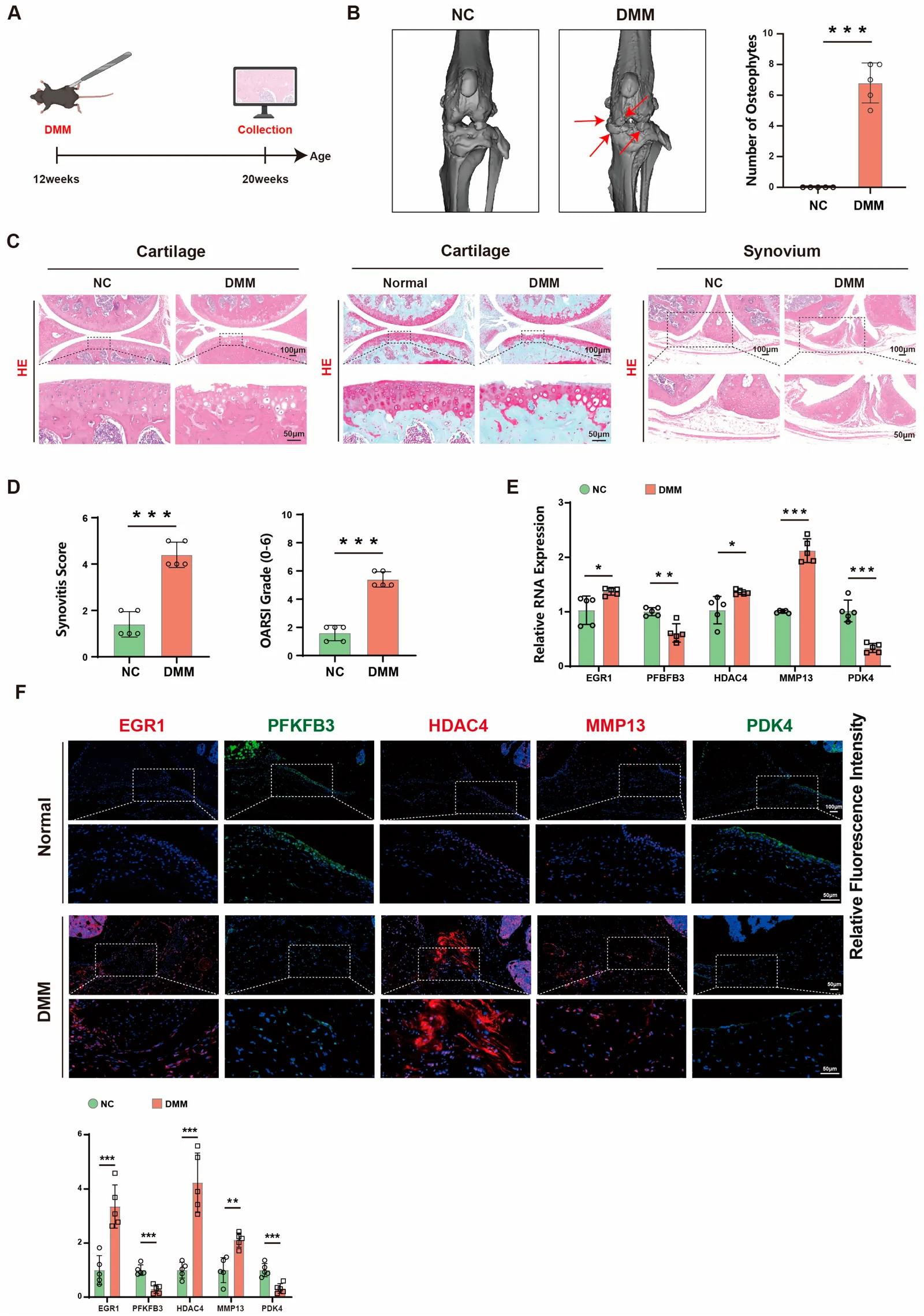
(**A**) Preparation, sampling, and experimental schematic diagram of the DMM mouse model. (**B**) Representative 3D micro-CT reconstruction scans and identification of osteophytes (red arrows) in DMM joints and sham-operated joints. (**C**) Comparison of representative HE staining results in cartilage and synovial tissue between DMM and sham surgery. (**D**) The staining results of sham operation samples and DMM samples were measured by the synovitis scoring and OARSI scoring systems. (**E**) qRT-PCR was used to detect the expressions of *EGR1*, *PFKFB3*, *HDAC4*, *MMP13* and *PDK4*. (**F**) Representative IF staining of *EGR1*, *PFKFB3*, *HDAC4*, *MMP13* and *PDK4*. Asterisks indicate statistical significance: * *p* < 0.05, ** *p* < 0.01, and *** *p* < 0.001.

According to the description in the experimental method, the microCT results of the knee joint tissue of DMM mice confirmed the quality of the OA model (Figure 15A,B). In the subsequent HE staining, cartilage tissues and synovial tissues from representative DMM joints and sham operation joints were selected, clearly showing cartilage degeneration and synovial injury in the DMM model (Figure 15C,D). The relative RNA expression levels of *EGR1*, *PFKFB3*, *HDAC4*, *MMP13* and *PDK4* genes in the sham operation group (NC) and the DMM group were detected by RT-qPCR (Figure 15E). The results showed that compared with the sham operation group (NC), the expressions of *EGR1*, *HDAC4* and *MMP13* in the DMM group were significantly increased (*p* < 0.05). The expressions of *PFKFB3* and *PDK4* were significantly decreased (*p* < 0.05), among which the expression differences of *MMP13* and *PDK4* genes were the most significant (*p* < 0.001), indicating that the establishment of the DMM model might have induced the upregulation of these genes’ expressions. The immunofluorescence experiment further verified the expression of the above-mentioned key genes at the protein level (Figure 15F). In normal tissues, the fluorescence intensities of *EGR1*, *HDAC4*, and *MMP13* are relatively weak, while those of *PFKFB3* and *PDK4* are relatively strong. In the DMM group, the fluorescence signals of these proteins showed differences. The results of relative fluorescence intensity analysis were consistent with those of RT-qPCR, both indicating that the expressions of each key gene in the DMM group were significantly different from those in the sham operation group (NC) (*p* < 0.01). This indicates that under the DMM model, these genes also show corresponding differences at the mRNA level and protein expression level.

## 4. Discussion

This study employed various bioinformatic analyses to explore the molecular mechanisms and potential biomarkers of OA. Initially, we retrieved and combined the GEO GSE82107 and GSE169077 datasets and then performed batch-effect correction to ensure data comparability. DEG analysis identified 80 significant DEGs, which, when intersected with 4592 known ARGs, revealed 21 ARDEGs. Using the STRING database, we identified 10 hub genes related to OA that were associated with acetylation. Subsequently, we applied LASSO regression to select six potential diagnostic biomarkers for OA: *EGR1*, *PFKFB3*, *HDAC4*, *MMP13*, and *ACADL*. The boxplots showed differences in the expression of these genes in OA samples compared with normal samples, whereas the results of the ROC curve analysis suggested a potential diagnostic value of these key genes within the combined GEO dataset. CIBERSORTx and ssGSEA revealed exploratory differences in immune cell infiltration between patients with OA and healthy controls, which should be interpreted with caution given the small sample size and the lack of FDR-adjusted significance. The results of the GO and KEGG enrichment analyses indicated that these hub genes were primarily involved in small-molecule metabolic processes, lipid biosynthesis, parathyroid hormone secretion, and apelin signaling. Finally, verification experiments on human tissues and mouse models demonstrated that mRNA levels and protein expression of *EGR1*, *HDAC4*, and *MMP13* were significantly up-regulated in OA tissues compared with the control group, while *PFKFB3* and *PDK4* were significantly down-regulated. This study, through the integration of bioinformatics analysis and experimental verification, systematically screened six acetylation-related differentially expressed genes and characterized their association patterns in OA, immune cell infiltration, and metabolic regulation, providing a basis for future mechanistic exploration. It should be emphasized that these findings describe correlational associations rather than acetylation-dependent regulatory mechanisms, which require functional experiments to validate.

Our PPI network analysis revealed that 12 out of the 21 ARDEGs have significant protein–protein interactions, forming two functionally distinct modules. The structural module is centered on *COL1A2* and connects *MMP13*, *EGR1* and *HDAC4*, reflecting the regulatory network of extracellular matrix homeostasis; the metabolic module consists of *PDK4*, *PCK1*, *PFKFB3* and *ACADL*, representing the core nodes of energy metabolism reprogramming. The modular organization pattern is highly consistent with the previous findings of OA network studies. Wang et al. identified 23 OA-related hub genes through WGCNA, and found that these genes are also clustered in functional modules such as cell adhesion, leukocyte activation, and inflammatory response. Similarly, Wang et al. also observed the core position of immune-related genes in the OA network, supporting the integration of the immune-metabolism-matrix degradation network in OA [8]. The structural module in our network is centered on *COL1A2*, connecting *MMP13*, *EGR1* and *HDAC4*. This topological structure is similar to the matrix degradation network observed in previous studies. Research indicates that *HDAC4* regulates chondrocyte hypertrophy by directly binding to Runx2 and inhibiting its transcriptional activity [40,41]. Runx2 is a key transcriptional regulator of *COL1A2* and *MMP13* [42]. Furthermore, the *HDAC4*-deficient mice exhibited chondrocyte hypertrophy, increased expression of *MMP13*, and premature mineralization of bones, further supporting the central role of *HDAC4* in maintaining chondral homeostasis. The application of the CytoHubba algorithm ensures the objectivity of the selection of hub genes. We adopted the consensus results of three topological algorithms (MCC, MNC, and Degree), and this multi-algorithm integration strategy significantly improved the reliability of hub gene identification. A single algorithm may produce false positives due to network noise, while the consensus of multiple algorithms can effectively reduce this risk. From the 10 hub genes, LASSO regression further selected 6 genes with non-zero coefficients (*EGR1*, *PFKFB3*, *HDAC4*, *MMP13*, *PDK4*, *ACADL*). This simplification process is based on statistical independence and predictive efficacy, and is consistent with the feature selection strategies in previous biomarker studies [43,44]. The metabolic module consists of *PDK4*, *PCK1*, *PFKFB3*, and *ACADL*, representing the core nodes of energy metabolism reprogramming. The discovery of this module is in line with the recent hotspots in OA metabolic research. Our PPI network directly links these metabolic genes with acetylation modifications, suggesting that acetylation may participate in OA metabolic reprogramming by regulating the activity of metabolic enzymes.

The gene correlation analysis revealed possible biologically significant co-expression patterns in OA, which reflect the functional coordination and competitive relationships among biological processes. The positive correlation between *MMP13* and *COL1A2* (r = 0.677) is generally a contradictory process, but this might suggest a malignant cycle of matrix remodeling in OA. *MMP13*, as the main collagen-degrading enzyme, when its activity increases, it leads to the breakdown of type I collagen. The broken collagen fragments can activate the TGF-β/Smad pathway through integrin signaling, thereby inducing the expression of *COL1A2* in fibroblasts [45]. This pathological cycle in OA may reflect that both the cartilage and synovial tissues are simultaneously subjected to mechanical stress and inflammatory stimulation. From the perspective of acetylation regulation, the promoter regions of the *MMP13* and *COL1A2* genes both contain multiple CpG islands and histone acetylation sites. The abnormal expression of *HDAC4* may simultaneously affect the chromatin accessibility of these two genes, leading to their coordinated dysregulation. The negative correlation between *ACADL* and *COL1A2* (r = −0.69) compatible with an untested hypothesis of metabolic resource competition, though alternative explanations cannot be excluded. The negative correlation between the downregulation of *ACADL* and the upregulation of *COL1A2* may reflect the transfer of metabolic substrates from energy production to biosynthesis. This metabolic reprogramming is similar to the Warburg effect and is an adaptive response of cells under stress conditions [46]. It is possible that maintaining this stress response over a long period in OA leads to pathological changes.

We found that in OA, naive B cells increased while resting mast cells decreased, although these differences did not remain significant after multiple-testing correction. Similarly, analysis of the single-cell maps of IPFP and synovial membranes also suggested an imbalance between immune activation and adaptive immune suppression in OA [47]. The increase in naive B cells may reflect the formation of new lymphoid follicles, which is consistent with the histological observation of ectopic lymphoid tissue in the synovium of OA [48]. It is notable that the decrease in resting mast cells rather than the increase in activated mast cells may reflect the depletion of the mast cell pool caused by chronic inflammation, and continuous inflammatory stimulation may lead to increased mast cell apoptosis or phenotypic drift. Compared with the limited changes in cell numbers suggested by CIBERSORTx, ssGSEA suggested extensive activation of immune functional pathways, including I/II type interferon response, macrophage function, checkpoint molecules, and T cell co-inhibition pathways. This stable but functionally activated pattern may reflect the characteristics of chronic low-grade inflammation progression in OA. From the perspective of acetylation regulation, the metabolic-function status of immune cells is dynamically regulated by histone acetylation dynamics. Acetyl-CoA, as the substrate for histone acetylation, its subcellular distribution directly determines the epigenetic state of inflammatory genes. The downregulation of *PDK4*/*PFKFB3* we observed may lead to the inhibition of fatty acid oxidation, promoting the acetylation of histones at the promoters of pro-inflammatory genes from mitochondria to cytoplasm.

The BP enrichment revealed significant alterations in “cytoplasmic translation”, “reproductive system development”, and “rhythmic processes”, suggesting the reactivation of the protein synthesis machinery and developmental programs. The enrichment of “nuclear speck” in the CC analysis holds particular significance: nuclear specks are storage sites for pre-mRNA splicing factors, and their functions are regulated by histone acetylation [49]. The nuclear-cytoplasmic shuttling of *HDAC4* [50] may affect the composition of nuclear specks and the efficiency of pre-mRNA splicing, providing a new perspective for understanding the role of acetylation in post-transcriptional regulation. MF analysis emphasizes “ECM structural components” and “collagen binding” activities, reinforcing the central role of the imbalance in matrix homeostasis. The PI3K-Akt signaling pathway is enriched in KEGG. Previous studies have shown that Akt activity is regulated by acetylation: SIRT1-mediated deacetylation of Akt enhances its kinase activity, while the acetylation effect of p300/CBP is the opposite [51,52]. In OA, the excessive activation of PI3K-Akt promotes the survival of chondrocytes but inhibits autophagy, leading to the accumulation of senescent cells and the production of SASP [53]. In the analysis of inter-sample pathway activity heterogeneity revealed by GSVA, the high upregulation of the EMT (epithelial-mesenchymal transition) pathway shares molecular programs with chondrocyte hypertrophic differentiation (Snail/Slug transcription factors, N-cadherin expression) [54], suggesting that OA chondrocytes acquire migratory and proliferative phenotypes. Upregulation of Angiogenesis and TGF-β signaling corresponds to synovial pannus formation and fibrosis, respectively [55,56].

Our validation experiment design followed the strategy of “from humans to animals, from mRNA to protein”, which enhanced the reliability of the findings. The triple verification using qRT-PCR, Western blot, and immunofluorescence showed highly consistent results: EGR1, HDAC4, and MMP13 were upregulated in OA, while *PFKFB3* and *PDK4* were downregulated. Notably, among the six LASSO-selected hub genes (*EGR1*, *PFKFB3*, *HDAC4*, *MMP13*, *PDK4*, and *ACADL*), we performed comprehensive experimental validation for five genes, while *ACADL* was not included in the experimental validation. This strategic decision was based on three carefully considered factors. First, in the ROC analysis of the 6 genes selected by LASSO, the AUC value of *ACADL* was the lowest and was less than 0.7. *ACADL* exhibited extremely low basal expression in chondrocytes and macrophages (<1 UMI per cell) in our single-cell analysis of the GSE216651 dataset, approaching the detection limit of single-cell sequencing technology, which raised concerns about the feasibility of reliable detection in traditional bulk tissue experiments. Second, the five validated genes (*EGR1*, *PFKFB3*, *HDAC4*, *MMP13*, and *PDK4*) already cover four critical functional modules central to OA pathogenesis: transcriptional regulation (*EGR1*), epigenetic regulation (*HDAC4*), energy metabolism (*PFKFB3*/*PDK4*), and extracellular matrix degradation (*MMP13*), providing a robust framework for understanding the multi-dimensional roles of acetylation-related genes in OA. Third, resource optimization considerations led us to prioritize genes with stronger differential expression patterns and clearer functional associations with acetylation modifications in the existing literature. The validation of these five genes through multiple experimental modalities (human synovial tissue qRT-PCR, Western blot, immunofluorescence, and DMM mouse model) and their cross-species conservation strongly supports our core conclusions regarding the involvement of acetylation-related gene networks in OA pathogenesis.

Notably, OA and lipid metabolism are closely related [57], and the chondrocytes of patients with OA often exhibit substantial lipid deposition [58]. While direct acetylation of *PDK4* has not been extensively characterized, *PDK4* expression is intimately linked to acetyl-CoA metabolism and PPAR pathway activation. *PDK4* plays a role in inactivating the pyruvate dehydrogenase complex through phosphorylation and is linked to lipid metabolism in skeletal muscle [59]. This inactivation inhibits the conversion of pyruvate to acetyl-CoA, thereby shifting the utilization of energy substrates from carbohydrates to lipids [60,61]. The results of this study corroborate those of previous research demonstrating reduced expression of *PDK4* in patients with OA. Moreover, *PDK4* also inhibits OA progression by activating the peroxisome proliferator-activated receptor pathway. *PDK4* overexpression can suppress apoptosis in chondrocytes induced by interleukin (IL)-1β, as well as the levels of inflammatory cytokines and the expression of ECM degradation proteins such as MMP3, MMP13, and a disintegrin and metalloproteinase with thrombospondin motifs 4 (ADAMTS-4) [62]. Song et al. proposed *PDK4* as a key gene responsible for resistance to ferroptosis. *PDK4* has a significant impact on cellular metabolism by blocking the oxidation of pyruvate that is dependent on pyruvate dehydrogenase, and this mechanism plays a crucial role in inhibiting ferroptosis [63]. Ferroptosis is a common phenomenon of cell death in OA, and ferroptosis in chondrocytes is associated with OA [64].

*PFKFB3* is a bifunctional enzyme that regulates glycolysis and has emerged as pivotal in immunology and metabolic research. *PFKFB3* allosterically activates the key rate-limiting enzyme in glycolysis, 6-phosphofructokinase-1, by regulating and maintaining the concentration of fructose-2,6-bisphosphate within the cell, thereby accelerating glycolysis [65]. In acetylation-related studies, acetylation of K472 of PFKFB3 affects its interaction with the nuclear importer importin α5, thereby regulating its localization in the cytoplasm and nucleus and affecting the cellular glycolytic process [66]. Qu et al. found that *PFKFB3* regulates glycolytic metabolism and alleviates endoplasmic reticulum stress in human OA cartilage. Overexpression of *PFKFB3* can improve the inhibited glycolysis process in OA cartilage, while also reducing the expression of ER stress-related genes such as *MMP13*. Furthermore, *PFKFB3* enhances chondrocyte viability, decreases caspase-3 activation, and promotes aggrecan and type II collagen expression, representing a potential target for OA prevention and treatment [67]. Xu et al. demonstrated improved articular cartilage appearance and physiological structure with highly organized chondrocytes and an abundant cartilage matrix in rats treated with harpagide (HPG) compared with the OA group. Furthermore, molecular docking results of HPG with key glycolytic factors indicated that HPG has good binding potential with *PFKFB3* [68].

*MMP13* has been reported to play a crucial role in OA progression. It belongs to the zinc-dependent MMP family and can degrade various ECM proteins [69]. The soluble collagenases MMP1, MMP8, and MMP13 are essential for this degradation process, with MMP13 being dominant [70]. *MMP13* expression is regulated by acetylation dynamics at both histone and non-histone levels. Recruitment of bromodomain-containing proteins (Brd3/Brd4) to acetylated chromatin is essential for cytokine-induced MMP13 transcription [71]. During OA development, *MMP13* is primarily responsible for the degradation of type II collagen in the articular cartilage. Additionally, *MMP13* degrades proteoglycans, type IV and IX collagen, osteopontin, and cartilage basement membrane glycoproteins in the cartilage matrix, all of which are integral components of the cartilage matrix [72]. MMP13 is a key factor in the pathological process of OA, and it initiates the degradation of matrix and collagen components, playing a critical role in OA development [73,74,75]. *MMP13* inhibitors could serve as novel therapeutic strategies for OA [76,77]. Recent studies have highlighted the key role of the TGF-β1/miR-4327/CBP/RUNX2 axis in *MMP13* expression, which is clinically relevant for the treatment of bone-related diseases [78]. Furthermore, many differentially expressed miRNAs in OA that play roles in OA pathology have been identified, with some of these miRNAs regulating *MMP13* expression [79].

*EGR1* is a C2H2-type zinc-finger protein. As a transcriptional regulator, EGR1 modulates the expression of target genes involved in cell proliferation, growth, and differentiation. An imbalance in chondrocyte proliferation, differentiation, apoptosis, and metabolism leads to OA onset. *EGR1* is subject to complex acetylation-mediated regulation at multiple levels. The *EGR1* promoter exhibits rapid, nucleosome-specific histone acetylation (H3K9ac, H3K27ac, H4K16ac) upon cytokine stimulation, preceding transcriptional activation [80]. Furthermore, *EGR1* protein itself is acetylated by p300/CBP, which stabilizes the protein and promotes its transcriptional activity [81]. The *EGFR* signaling pathway plays a significant role in regulating chondrocyte growth and metabolism. In OA, *EGR1* has been reported to suppress collagen-type II alpha 1 (COL2) expression, induce cartilage matrix degradation enzymes (MMP9 and MMP13) expression, enhance hypertrophy-associated gene (COLX) expression, and induce chondrocyte degeneration and hypertrophy [82]. In animal studies, a rat model of OA exhibited higher *EGR1* expression and increased angiogenesis in the cartilage than sham-operated rats [83]. Numerous bioinformatics studies have identified *EGR1* as a key gene in OA, and Fei et al. proposed EGR1 as an upstream regulator of the gene expression signature in the synovial tissue of OA [84,85]. Importantly, one study reported a significant reduction in cartilage degeneration and knee joint pain in mice overexpressing the *EGFR* pathway, demonstrating the feasibility of targeting the *EGFR* signaling using nanotechnology for treating OA [86].

HDAC4 is primarily distributed in the brain, muscle, and cartilage and plays a significant role in the pathological processes of OA. HDAC4, a class IIa HDAC, is a zinc-dependent enzyme that removes acetyl groups through its zinc-containing catalytic domain. It plays an important role in regulating gene expression and cell growth, survival, and proliferation [87]. Changes in HDAC activity, expression, and distribution can lead to OA development. HDAC inhibitors (HDACis) can protect chondrocytes and prevent cartilage damage [88,89]. Notably, animal studies have provided compelling evidence of the role of *HDAC4* in OA. Gu et al. showed that *HDAC4* in OA is involved in regulating the activating transcription factor 4 and C/EBP homologous protein signaling pathway, which is crucial for inhibiting chondrocyte apoptosis and reducing cartilage damage [90]. Wen et al. demonstrated that intra-articular injection of the HDACi panobinostat protected against cartilage degeneration in rats and alleviated joint pain by modulating *HDAC4* [91]. Additionally, *HDAC4* suppresses myocyte-specific enhancer factor 2C and RUNX2, thereby controlling chondrocyte hypertrophy and endochondral ossification [41,92]. Collectively, these studies indicate that *HDAC4* plays a potential role in OA pathogenesis, which was also confirmed by the results of the present study.

This study also has some limitations. This was a retrospective study and lacked new clinical samples and data. Moreover, the sample size may be insufficient and may be subjected to selection bias because of the reliance on public databases, restricting the generalizability and reliability of our findings. Our bioinformatics analysis combined data from synovial membrane and articular cartilage, two tissues with distinct cellular compositions, gene expression profiles, and pathophysiological roles in OA. This cross-tissue integration was performed as an exploratory analysis and cannot replace tissue-specific analyses. The integration of synovial membrane and cartilage datasets, while common in OA bioinformatics studies, may mask tissue-specific gene expression patterns. Future studies should employ tissue-stratified analyses and spatial transcriptomics to dissect cell-type-specific dysregulation. The immune-infiltration analysis is underpowered due to the small sample size of the combined GEO dataset, and none of the cell-type differences remained statistically significant after Benjamini–Hochberg correction for multiple testing. The observations regarding mast-cell depletion, B-cell activation, and interferon signaling are exploratory and speculative, requiring validation by flow cytometry, immunohistochemistry, or spatial transcriptomics in future studies. The LASSO-ROC signature was developed and tested on the same small dataset without independent external validation. The AUC values, while promising, are likely optimistic and require confirmation in prospective cohorts with clinically diagnosed OA patients and appropriate controls. The single-cell analysis was employed primarily for orthogonal validation of differential expression patterns rather than comprehensive cell-type-specific disease dysregulation. Future studies should exploit disease-state-stratified single-cell analyses and spatial transcriptomics to dissect the cellular localization and functional roles of these hub genes in the OA joint microenvironment. Additionally, among the six hub genes identified by LASSO regression (*EGR1*, *PFKFB3*, *HDAC4*, *MMP13*, *PDK4* and *ACADL*), experimental validation was completed for only five genes, with *ACADL* excluded because its AUC value in its ROC analysis was relatively low, its extremely low expression levels in relevant cell types and technical feasibility constraints. This omission may affect the completeness of our mechanistic network presentation, particularly regarding the metabolic module of the PPI network where *ACADL* plays a crucial role in fatty acid oxidation. The negative correlation we observed between *ACADL* and *COL1A2* (r = −0.69) suggests potential metabolic competition between energy production and matrix synthesis, a hypothesis that remains to be experimentally validated. Future studies should also validate *ACADL* through metabolomics collaborations, gene overexpression/knockout models, or targeted metabolite analysis, etc. Moreover, this study did not fully account for the multifactorial aspects of OA, including the interplay between genetics, environment, and lifestyle factors, and long-term follow-up data to assess disease progression and treatment efficacy were lacking. There are several limitations in both histological and animal studies. Only five mice were included per group with the contralateral knee as control; although this paired design reduces variability, systemic effects from DMM surgery may influence the contralateral limb. Although cartilage degeneration and synovitis were confirmed by HE staining and quantitatively evaluated using the OARSI and synovitis scoring systems (Figure 15C,D), these histological assessments were based on a relatively small number of joints and may be subject to scoring subjectivity, and the number of mice per group was limited, limiting cartilage and synovial pathology evaluation. Our findings are correlational; the altered gene expression in OA joints does not establish causal roles. Functional studies (e.g., gene manipulation, pharmacological interventions) are needed to validate their significance. These limitations will be addressed in future investigations. It is critical to emphasize that our study describes correlational associations between the expression of acetylation-related genes and OA status, not acetylation-dependent regulatory mechanisms. The differential expression of *HDAC4*, *EGR1*, *PFKFB3*, *PDK4*, *MMP13*, and *ACADL* in OA tissues does not demonstrate that altered acetylation of these proteins or their substrates directly drives OA pathogenesis. Functional experiments—including acetylome profiling, site-directed mutagenesis of acetylation sites, and rescue experiments with acetylation mimetics—are required to establish causal roles. Finally, we did not perform gain-of-function, loss-of-function, or rescue experiments to establish causal roles for the identified genes in OA. CRISPR-mediated gene editing, siRNA knockdown, or overexpression studies in primary chondrocytes/synoviocytes, as well as conditional knockout mouse models, are essential next steps to validate the functional significance of *HDAC4*, *EGR1*, *PFKFB3*, *PDK4*, *MMP13*, and *ACADL* in OA pathogenesis. Future research should address these limitations by increasing the sample size, employing multiple model systems for validation, considering a broader range of confounding factors, and assessing the impact of interventions.

## 5. Conclusions

This study involved comprehensive bioinformatic analysis to identify ARDEGs in OA and utilized PPI networks, LASSO regression analysis, and other statistical methods to identify six potential biomarker genes: *HDAC4*, *EGR1*, *PFKFB3*, *PDK4*, *MMP13*, and *ACADL*. Finally, experiments on mouse OA tissues and human OA tissues demonstrated differences in mRNA levels and protein expression of five of these potential biomarker genes. Moreover, our results highlighted their potential involvement in OA progression and their putative functions in immune cell infiltration, metabolic processes, and signaling pathways. These findings offer hypotheses regarding the molecular mechanisms underlying OA and suggest candidate targets for future mechanistic exploration and therapeutic development, pending functional validation of acetylation-dependent regulation and causal roles. Meanwhile, future studies involving large sample sizes are essential to validate our findings in vivo models and comprehensively assess specific therapeutic interventions.

## Data Availability

The datasets analyzed in this study were obtained from publicly available repositories. The microarray data are available in the NCBI Gene Expression Omnibus (GEO) database under accession numbers GSE82107 and GSE169077, and the single-cell RNA-seq data are available under accession number GSE216651 (https://www.ncbi.nlm.nih.gov/geo/). Access date: 6 August 2026.

## Supplementary Materials

The following supporting information can be downloaded at: https://www.mdpi.com/article/10.3390/biomedicines14081806/s1, Table S1: PCR primers sequence. Table S2: GO-Results. Table S3: KEGG-Results. Table S4: GSVA-Results.

## Abbreviations

The following abbreviations are used in this manuscript:

ACADL	Acyl-CoA dehydrogenase long chain
ADAMTS-4	a Disintegrin and metalloproteinase with thrombospondin motifs 4
ARDEGs	Acetylation-related differentially expressed genes
ATF3	Activating transcription factor 3
AUC	Area under the curve
BASP1	Brain abundant, membrane attached signal protein 1
B3GALNT1	Beta-1,3-N-acetylgalactosaminyltransferase 1
BP	Biological processes
Brd	Bromodomain
BCA	Bicinchoninic acid
CBP	CREB-binding protein
CC	Cellular components
CD4	Cluster of differentiation 4
CD52	Cluster of differentiation 52
CGA	Glycoprotein hormones, alpha polypeptide
COL1A2	Collagen-type I alpha 2 chain
CPM	Counts per million
CRIP1	Cysteine-rich protein 1
CTNS	Cystinosin, lysosomal cystine transporter
DAPI	4′,6-diamidino-2-phenylindole
DEGs	Differentially expressed genes
DMM	Destabilisation of the medial meniscus
ECL	Enhanced chemiluminescence
ECM	Extracellular matrix
EGR1	Early growth response 1
EGFR	Epidermal growth factor receptor
EMT	Epithelial-Mesenchymal Transition
FUT4	Fucosyltransferase 4
GEO	Gene Expression Omnibus
GO	Gene Ontology
GSVA	Gene Set Variation Analysis
HDAC	Histone deacetylase
HDACs	Histone deacetylases
HDAC3	Histone deacetylase 3
HDAC4	Histone deacetylase 4
HDACi	Histone deacetylase inhibitors
HE	Hematoxylin and Eosin
HPG	Harpagide
IF	Immunofluorescence
IL-1β	Interleukin-1β
IPFP	Infrapatellar fat pad
ITGB2	Integrin subunit beta 2
KEGG	Kyoto Encyclopedia of Genes and Genomes
LASSO	Least absolute shrinkage and selection operator
log2FC	Log2 fold change
logFC	Log fold change
MCC	Maximal Clique Centrality
MF	Molecular functions
MMP13	Matrix metallopeptidase 13
MNC	Maximal Neighborhood Component
MSigDB	Molecular Signatures Database
NC	Normal control
OARSI	Osteoarthritis Research Society International
OA	Osteoarthritis
PCA	Principal Component Analysis
PCK1	Phosphoenolpyruvate carboxykinase 1
PDK4	Pyruvate dehydrogenase kinase 4
PFKFB3	6-phosphofructo-2-kinase/fructose-2,6-bisphosphatase 3
P3H2	Prolyl 3-hydroxylase 2
PIGA	Phosphatidylinositol glycan anchor biosynthesis class A
PPI	Protein–protein interaction
PVDF	Polyvinylidene fluoride
qRT-PCR	Quantitative reverse transcription-polymerase chain reaction
ROC	Receiver operating characteristic
Runx2	Runt-related transcription factor-2
SASP	Senescence-associated secretory phenotype
SDS-PAGE	Sodium dodecyl sulfate-polyacrylamide gel electrophoresis
SELL	Selectin L
SEM	Standard error of the mean
SGK1	Serum/glucocorticoid regulated kinase 1
SIRT1	Sirtuin 1
ssGSEA	Single-sample Gene Set Enrichment Analysis
SULF1	Sulfatase 1
TAC1	Tachykinin precursor 1
TGF-β	Transforming growth factor-beta
TPPP3	Tubulin polymerization promoting protein family member 3
TWIST1	Twist family bHLH transcription factor 1
t-SNE	T-distributed Stochastic Neighbor Embedding
UMAP	Uniform Manifold Approximation and Projection
UMI	Unique Molecular Identifier
WB	Western blot
WGCNA	Weighted Gene Co-expression Network Analysis
WT	Wild type
